# Confinement Effects on Reorientation Dynamics of Water Confined within Graphite Nanoslits

**DOI:** 10.1021/acs.jpcb.4c03898

**Published:** 2024-09-23

**Authors:** Chi-Wei Wang, Yu-Wei Kuo, Jing-Rong Zeng, Ping-Han Tang, Ten-Ming Wu

**Affiliations:** Institute of Physics, National Yang Ming Chiao Tung University, Hsinchu 300, Taiwan

## Abstract

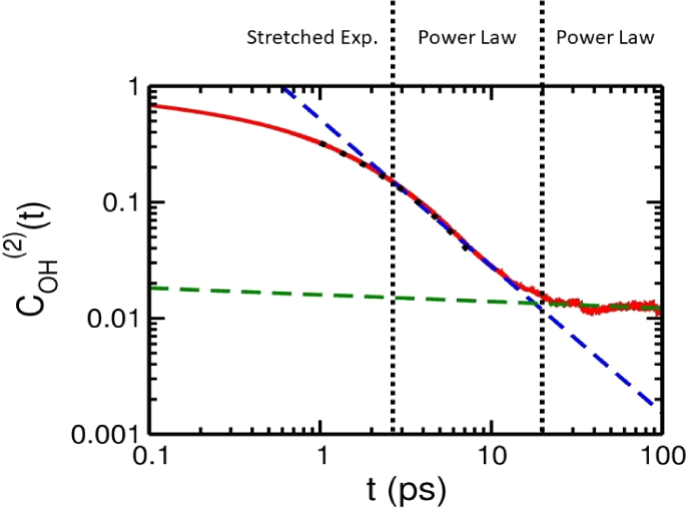

Molecular dynamics simulations were used to investigate the reorientation dynamics of water confined within graphite nanoslits of size less than 2 nm, where molecules formed inner and interfacial layers parallel to the confining walls. Significantly related to molecular reorientations, the hydrogen-bond (HB) network of nanoconfined water therein was scrutinized by HB configuration fractions compared to those of bulk water and the influences on interfacial-molecule orientations relative to a nearby C atom plate. The second-rank orientation time correlation functions (OTCFs) of nanoconfined water were calculated and found to follow stretched-exponential, power-law, and power-law decays in a time series. To understand this naïve behavior of reorientation relaxation, the approach of statistical mechanics was adopted in our studies. In terms of the orientation Van Hove function (OVHF), an alternative meaning was given to the second-rank OTCF, which is a measure of the deviation of the OVHF between a molecular system and free molecules in random orientations. Indicated by the OVHFs at related time scales, the stretched-exponential decay of the second-rank OTCF resulted from molecules evacuating out of HB cages formed by their neighbors. After the evacuations, the inner molecules relaxed at relatively fast rates toward random orientations, but the interfacial molecules reoriented at slow rates due to restrictions by hydrophobic interactions with graphite walls. The first power-law decay of the second-rank OTCF was attributed to the distinct relaxation rates of inner and interfacial molecules within a graphite nanoslit. When the inner molecules were completely random in orientation, the second-rank OTCFs changed to another power law decay with a power smaller than the first one.

## 1 Introduction

Water reorientation has attracted many researchers and plays an important role in numerous chemical reactions and mechanisms in liquid water and aqueous solutions,^1,2^ involving proton transfer,^3^ proton transport,^4,5^ the Grotthuss mechanism,^6^ etc. The reorientation dynamics of water molecules in liquid water and aqueous solutions is strongly related to the rearrangement of the hydrogen-bond (HB) network via the continual forming and breaking of HBs,^7^ exchanging HB acceptors by a large-amplitude jump,^8,9^ collective HB network dynamics,^10−12^ and vibrational dephasing.^13,14^

Water confined within nanopores is quite different in structure and dynamics from bulk water due to the confinement as well as molecular interactions with confining walls.^15−17^ Understanding the structure and dynamics of nanoconfined water is important to heterogeneous catalysis,^18^ biological channels in cell membranes,^19^ and technological applications such as seawater desalination^20−22^ and nanodevice fabrications.^23^ In the study of the confinement effects on molecular reorientations, water was usually confined within reverse micelles^24−28^ and silica nanopores,^29−33^ where the reorientation dynamics of confined water was measured. Reverse micelles, akin to biological systems, have soft interfaces, typically in spherical, ellipsoidal, or rod-like geometry.^34^ Silica glasses, with low background signals on optical detections, possess nanopores with rigid confining walls with roughness and containing silanol groups, which make pore surfaces hydrophilic and show polarity.^35^ Within these nanopores, confined water displayed much slower reorientation dynamics as compared with bulk water, where the relaxations were found to be nonexponential with debates upon stretched-exponential or power-law decay at different time scales.^36−38^ Besides the long-time reorientation relaxation, the second-rank orientation time correlation functions (OTCFs) of nanoconfined water exhibited a persisting residue, which has been observed by experiments^24,29^ and simulations^39^ for a long time, but a fundamental explanation for the persisting residual is still elusive.

The reorientation dynamics of nanoconfined water essentially rely on features of the confining walls of the container: hydrophobic or hydrophilic properties, surface topography and polarity, interfacial roughness,^40−43^ etc., as the size of the water container narrows down to nanoscales. In this paper, to address the debates described above, we used molecular dynamics (MD) simulations to study the reorientation dynamics of water confined within graphite nanoslits of size less than 2 nm, where the size of the graphite/graphene slit pore was controllable in fabrication and the confining walls were flat in topography, without patchiness, and free from local net charges.^23^ The interactions between water and graphite/graphene are hydrophobic and characterized as weak van der Waals forces. Within the graphite/graphene slits of size less than 2 nm, many intriguing dynamics of nanoconfined water with respect to bulk water were observed by experiments and simulations, including a fast flow rate^44^ and a great enhancement in shear viscosity, which exhibited an oscillatory behavior with the slit size.^45^

Calculated by our MD simulations, the second-rank OTCFs of water confined within graphite nanoslits were found to relax in a time profile of stretched-exponential, power-law, and power-law decay. To understand this naïve behavior of reorientation relaxation, the orientation Van Hove function (OVHF) was defined similarly to the self-part Van Hove function for translational motions.^46,47^ In relation to the OVHF, an alternative meaning was given to the second-rank OTCF, which is a measure of the deviation of the OVHF between a molecular system and free molecules in completely random orientations. In terms of the OVHFs, the naïve behavior of the second-rank OTCFs of nanoconfined water was explained, and their long-time residues resulted from interfacial molecules, which were restricted in reorientation due to hydrophobic interactions with a nearby confining wall of graphite.

The rest of the paper is organized as follows: Section 2 describes our MD simulations and the structures of nanoconfined water within graphite nanoslits. The HB network of nanoconfined water is analyzed in comparison to bulk water. In Section 3, the orientations of interfacial molecules relative to a nearby C atom plate are depicted and the intralayer-HB network of the layer is presented. In Section 4, the second-rank OTCFs of nanoconfined water at different time regimes are presented and fitted using different functions, and their behaviors are explained in terms of the OVHFs of confined water and layers within a graphite nanoslit. Our conclusions are given in Section 5.

## 2 Layer Structure and H-Bond Network

Presented in our prior work,^48^ a graphite nanoslit consists of two parallel C atom plates in AA-stacking on each side, where the slit geometric width *h* is the distance between the two C-atom inner plates with parallel directions satisfied with periodic boundary conditions. In three slit systems of *h* = 20, 15, and 10 Å, water molecules of number *N* = 1408, 1080, and 720 were, respectively, confined within each nanoslit such that the average water mass density ρ_geo_ between two inner plates of a nanoslit was nearly 1.015–1.038 g/cm^3^, which is close to that of liquid water under ambient conditions. However, repelled by hydrophobic interactions from C atom plates, water molecules were constrained within an effective slit width *h*_eff_ of a nanoslit, which was estimated to reduce about 3.22 Å from *h*, so that the effective mass density ρ_eff_ within the constrained region was near 1.21, 1.32, and 1.53 g/cm^3^ for *h* = 20, 15, and 10 Å, respectively, with external forces supposed to apply on both sides of a nanoslit to stabilize the slit system.^49^

In our model, TIP4P/2005 rigid water molecules were used,^50^ and the interactions between water and graphite were described by the Lennard-Jones (LJ) potentials between O–C atomic pairs, with the LJ parameters of O–O, C–C, and O–C atomic pairs referred to in ref (48), where our MD simulations are given in detail therein. Obtained from our MD simulations, side views of water molecules confined within graphite nanoslits are presented in Figure S1, indicating that water molecules formed a layered structure parallel to the C atom plates, with the layer boundaries determined at the minima of the O atom *z*-density profile shown in Figure 1 of ref (48). Within the nanoslit of *h* = 20 Å, there were six layers, referred as outer, next, and inner layers in distance from a nearby C atom plate. The nanoslits of *h* = 15 and 10 Å involved four and three layers, respectively, referred as outer and inner ones, with the inner layer occupied at the slit central region, where some fluctuations were found in the O atom z-density profile of *h* = 15 Å in comparison with the results of the SPC/E water model shown in Figure S2a. Within a nanoslit, the outer layer had a thickness decreasing with the slit width *h* and, therefore, a significant increase in its layer mass density.

**Figure 1:**
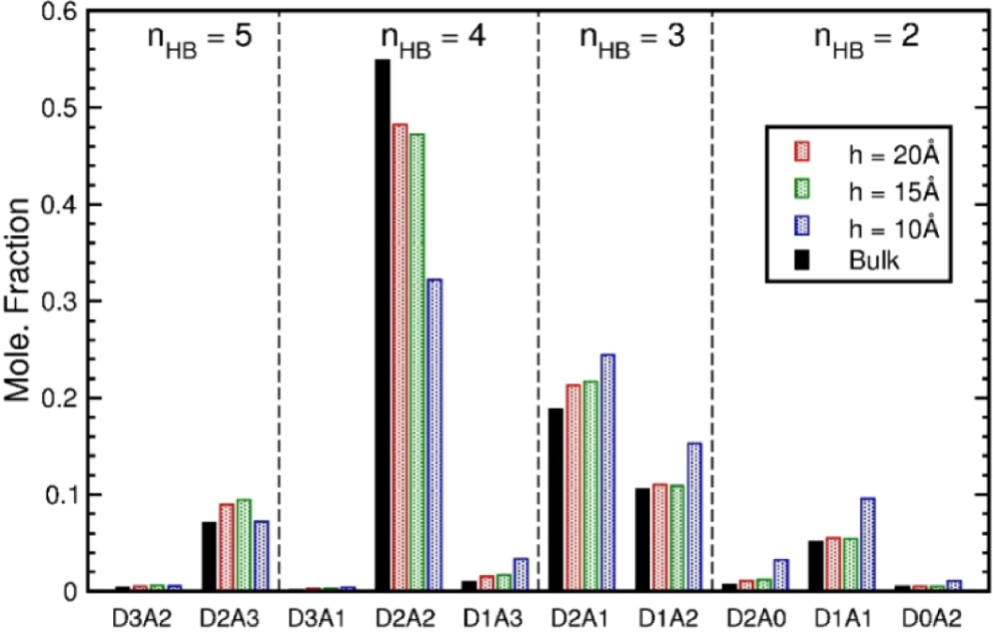
Fractions of HB configuration calculated for water molecules confined within graphite nanoslits. The fractions are the ratios of D*n*A*m* molecules with respect to all confined molecules. For a nanoslit of width *h*, the fractions of *n*_HB_ between 2 and 5 are shown by bars of the same symbol and are normalized to one. The black bars are the results of TIP4P/2005 liquid water.

Indicated by the lateral O–O radial distribution functions *g_xy_*(*r*) shown in Figure 2a of ref (48), nanoconfined water of *h* = 20 and 15 Å was in liquid-like states, where *g_xy_*(*r*) of all layers approached unity at long distances, whereas nanoconfined water of *h* = 10 Å was in solid-like states, where *g_xy_*(*r*) of outer and inner layers exhibited strong oscillations with slow decays. The assignments of the thermodynamic state for the three nanoconfined water were supported by their lateral translational diffusion coefficients,^48^ which were comparable to the value of liquid water for liquid-like nanoconfined waters but had a reduction in 1 order of magnitude for solid-like nanoconfined water. According to their effective mass densities, the thermodynamic assignments of the three nanoconfined water were consistent with the phase diagram of water confined within hydrophobic nanoslits.^51^

**Figure 2:**
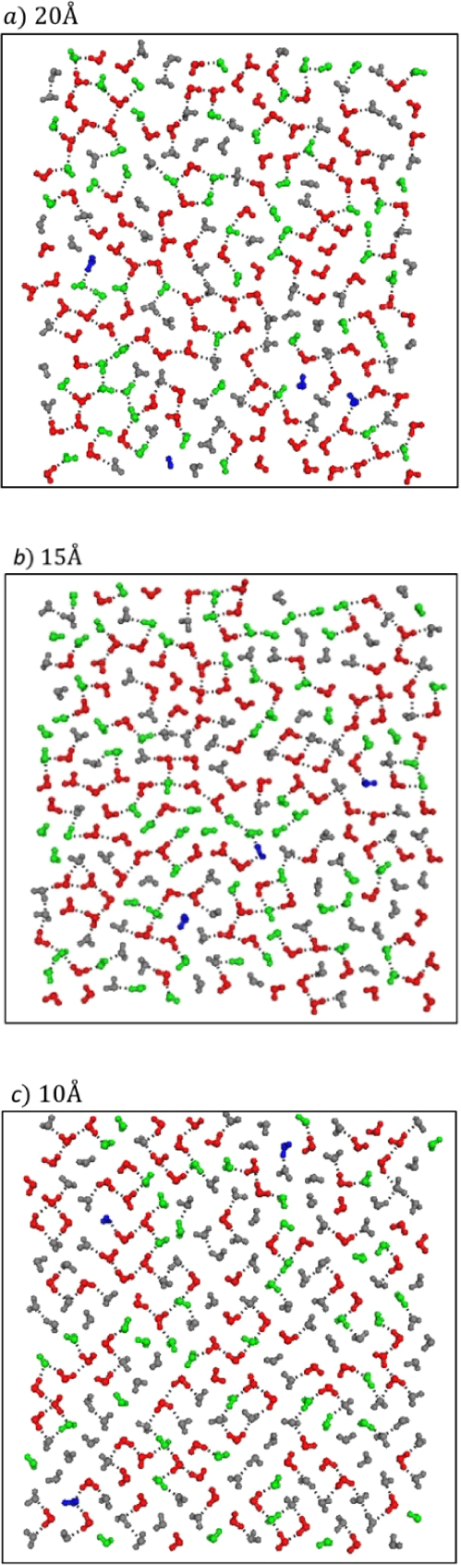
Top view of the intralayer HB network of interfacial molecules within a graphite nanoslit of width 20 Å (a), 15 Å (b), and 10 Å (c). Molecules are specified as parallel-like (red), vertical-like (green), dangling −OH (blue), and others (gray). The dotted lines indicate HBs between molecules. The configurations were generated from our MD simulations.

Listed in Table 2 of ref (48), the HB number *n*_HB_ of a confined molecule depends on the slit width and the layer that the molecule is embedded in, where the geometric HB definition was used.^52^ Averaged over all confined molecules within nanoslit, nanoconfined water had *n*_HB_ on average less than bulk water, with *n*_HB_ ≈ 3.69 for TIP4P/2005 liquid water. For liquid-like nanoconfined water, an outer layer had lower *n*_HB_ but the next and inner layers had *n*_HB_ comparable to bulk water, which is consistent with the reported results.^53^ In solid-like nanoconfined water, the outer and inner layers had *n*_HB_ ≈ 3.325 and 3.434, respectively, lower than that of liquid water. Due to the layered structure, the HB network of nanoconfined water can be characterized with intralayer and interlayer HB numbers of every layer therein, where the donor–acceptor molecular pair is located in the same layer or belongs to two adjacent layers, respectively. As nanoconfined water changed from liquid-like to solid-like states, an outer layer had a decrease in the intralayer HB but an increase in the interlayer HB.^48^

The HB network of nanoconfined water was analyzed by the fractions of HB configurations, denoted as D*n*A*m* for molecules having *n* donating and *m* accepting HBs within a neighborhood with the O–O distance less than 3.5 Å, where the HB configuration of a molecule significantly influences its short-time reorientation.^54^ The HB configuration fractions of nanoconfined water within a graphite nanoslit are presented in Figure 1, in reference to the results of bulk water.^55^ Though the fractions of HB configuration depend on the water model and the nanoslit system investigated,^56^Figure 1 displays general features for the variations of HB configuration with the slit width of nanoconfined water. First, the fraction of D2A2, the most stable local structure in bulk water, was reduced with the slit width, whereas the fractions of molecules with two or three HBs increased instead, which resulted from the slit confinement destroying the formation of the tetrahedral structure in nanoconfined water. In addition, nanoconfined water contained more fractions of bifurcated HB configurations, such as D2A3 and D1A3, with one OH-group connecting two acceptors,^54,57^ due to the layered structure within a nanoslit. Attentions were paid to the nanoslit of width 10 Å, where the D2A2 fraction decreased significantly and the fractions of other HB configurations increased substantially, indicating a broken HB network associated with solid-like nanoconfined water.

On HB dynamics, the fluctuation of an HB between a molecular pair can be measured by time correlation functions *C*_s_(*t*) and *C*_r_(*t*), where *C*_s_(*t*) is the surviving probability of an HB that remains intact up to time *t* without breaking and *C*_r_(*t*) is the probability of a molecular pair still intact an HB at time *t*, during which the HB may break and reform.^57^Figure S3 shows *C*_s_(*t*) and *C*_r_(*t*) calculated for the nanoconfined waters within graphite nanoslits, as compared with those of TIP4P/2005 bulk water. As indicated by the results therein, both *C*_s_(*t*) and *C*_r_(*t*) of nanoconfined water decay faster than the corresponding functions of bulk water, where the narrower the nanoslit size is, the fast the correlation functions decay. The lifetime τ_HB_ of an HB was estimated by the time at *C*_s_(τ_HB_)= 0.5, where the slit confinement made the τ_HB_ of nanoconfined water near 20–10 fs but the τ_HB_ of bulk water was near 100 fs due to its tetrahedral structure. For nanoconfined waters of *h* = 20 and 15 Å, and bulk water, a hump appeared in *C*_r_(*t*) near 50 fs, indicating the reformation of an HB after breaking.^57^ However, no such hump was observed in *C*_r_(*t*) of nanoconfined water of *h* = 10 Å due to its broken HB network.

## 3 Orientation of the Interfacial Molecule

Within a graphite nanoslit, an outer layer is the interface of nanoconfined water with a gap of atomic-scale distance from a C atom plate due to hydrophobic interactions. The HB partner of an interfacial molecule essentially depends on its orientation relative to the nearby C atom plate,^42^ and the HB network of an interfacial layer has a significant influence on its in-plane structure. Relative to a nearby C atom plate, the orientations of interfacial molecules can be described by two solid-angle distributions, *P*_N_(θ) and *P*_OH_(θ), as shown in Figure S4a,b, calculated with unit vector *ê*_N_ normal to the molecular plane and *ê*_OH_ along one OH-group of a molecule, respectively. Indicated by the solid-angle distributions, the interfacial molecules within a graphite nanoslit had two major orientations, parallel-like and vertical-like, where the parallel-like molecules had their molecular planes almost parallel to the C atom plate, and the vertical-like molecules had one OH-group pointing away from the plate, with the OH-group possibly connecting an HB with a molecule in the next layer, as shown in Figure S5. The two major orientations of interfacial molecules are consistent with previous reports for water near a hydrophobic flat interface.^42,58^ Besides the two orientations, a few interfacial molecules had one OH-group dangling in the gap between water and graphite and were referred as dangling −OH molecules. The three kinds of interfacial molecules relative to a C atom plate are shown in the inset of Figure S4b. For liquid-like nanoconfined water within a graphite nanoslit, nearly 45% of interfacial molecules were parallel-like, 27–28% were vertical-like, but 1 or 2% were dangling −OH molecules. For nanoconfined water within the nanoslit of *h* = 10 Å, the fractions of parallel-like and vertical-like interfacial molecules reduced to about 40 and 18–17%, respectively, while no change was found in the fraction of dangling −OH molecules. The significant reduction in the fraction of vertical-like molecules within the nanoslit of *h* = 10 Å was attributed to a strong squeeze on nanoconfined water by the confinement causing an extremely anisotropic environment on the interfacial layer, which has a narrower width of 2.14 Å as compared with 2.86 and 2.75 Å of nanoslits of *h* = 20 and 15 Å, respectively,^48^ so that the OH-groups connecting the inner layer were forced to deviate from the normal direction of confining wall.

Figure 2 presents examples of the intralayer-HB network of an interfacial layer with molecular orientations specified. As indicated by the figures, most interfacial molecules have two or three intralayer HBs; this situation is particular for solid-like nanoconfined water. As indicated by HB configurations of layers shown in Figure S6, an interfacial layer had a D2A2 fraction less than the inner layer due to the restriction from the nearby C atom plates on the formation of the tetrahedral structure; however, the interfacial molecules had fractions of two or three HBs more than bulk water. Hence, the in-plane structure of an interfacial layer changed from a tetrahedral structure and collinear sets of triplet molecules to square and rhombic structures, as nanoconfined water transformed from liquid-like to solid-like states.^48,59,60^

## 4 Reorientation Dynamics

The second-rank OTCFs of a molecular liquid are defined as
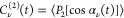
1with cos α_ν_(*t*) = *ê*_ν_(*t*)·*ê*_ν_(0), where *ê*_ν_(*t*) is a unit vector fixed to a molecule as observed at time *t* in the lab frame, *P*_2_(*x*) = (3*x*^2^ – 1)/2 is the second Legendre polynomial, the angular brackets denote an ensemble average over molecules, and α_ν_(*t*) is the angular displacement of *ê*_ν_(*t*) from its initial orientation *ê*_ν_(0) at *t* = 0. *ê*_OH_(*t*) and *ê*_B_(*t*) were considered here, where *ê*_B_(*t*) is along the molecular dipole, which bisects the HOH bond angle of a water molecule. For liquid water, *C*_OH_^(2)^(*t*) is proportional to the rotational anisotropy function measured by time-resolved IR pump–probe experiments,^61^ and its relaxation time can be obtained by NMR.^62,63^*C*_B_^(2)^(*t*) is related to spectroscopies of Raman scattering, light scattering, and fluorescence depolarization.^64^

Another quantity to characterize molecular reorientations in liquids is the orientation Van Hove function,^46^ which is defined as
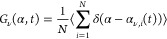
2where α_ν,*i*_(*t*) is the angular displacement α_ν_(*t*) of molecule *i*, with *i* running over molecules of a total *N*. *G*_ν_(α,*t*) is an angular distribution corresponding to the self-part Van Hove function of translational motions^47^ and is normalized over a range from 0 to π. In terms of *G*_ν_(α,*t*) defined in eq 2, an ensemble average of (cos α_ν_(*t*)^2^) is formulated as
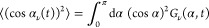
3By changing to a new variable *x* = cos α, ⟨(cos α_ν_(*t*))^2^⟩ is the second moment of a function *Q*_ν_(*x*,*t*) ≡ *G*_ν_(α,*t*)/sin α.

Take an example from free water molecules in a gas phase, where molecular orientations are completely random, which means that their molecular unit vectors at an instant are uniformly distributed on the surface of a unit sphere. In dynamics, the unit-vector orientation trajectory of a free molecule is expected to go around the whole surface of a unit sphere if time is long enough; this is referred to the ergodic theory in statistical mechanics.^65^ The OVHF *G*_un_(α) of free molecules at equilibrium is independent of time and simply proportional to sin α due to the Jacobin of spherical polar coordinates. According to eq 3, the average of (cos α_ν_(*t*)^2^) with respect to *G*_un_(α) gives ⟨(cos α_ν_(*t*))^2^⟩_un_ = 1/3, which is the square of the root of *P*_2_(*x*) = 0. Thus, the second-rank OTCF defined in eq 1 can be rewritten as
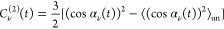
4This equation indicates that *C*_ν_^(2)^(*t*) is a measure of the deviation of *G*_ν_(α,*t*) at time *t* from *G*_un_(α). By tracing the orientation of a molecule, *C*_ν_^(2)^(*t*) indicates the coverage of its *ê*_ν_(*t*) trajectory on a unit-sphere surface during a process as molecular reorientations in a system become as random as possible.

### 4.1 Short Time Scales

**Figure 3:**
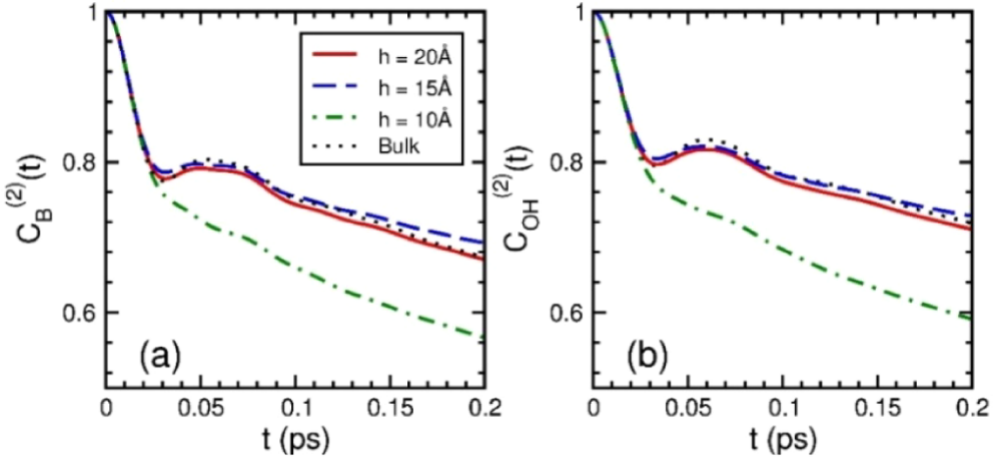
Second-rank OTCFs of nanoconfined water at short time scales: (a) *C*_B_^(2)^(*t*) and (b) *C*_OH_^(2)^(*t*). The OTCFs were obtained from MD simulations. The red solid, blue dash, and green dot-dashed lines are for nanoslits of width 20 Å, 15 Å, and 10 Å, respectively. The black dotted lines are for TIP4P/2005 liquid water under ambient conditions.

Figure 3a,b show the second-rank OTCFs at short times evaluated with *ê*_B_(*t*) and *ê*_OH_(*t*) for nanoconfined water, respectively. Up to 0.2 ps, the OTCFs of liquid-like nanoconfined water behaved similarly as that of bulk water by displaying a fast Gaussian decay, followed by a rebound within 0.1 ps and, then, changing to a slow decay, with the rebound of *h* = 15 Å, somewhat higher in magnitude than that of *h* = 20 Å, where the rebound of liquid water was resulted from liberations of molecules within HB cages formed by their neighbors.^14,54,66^ On the contrary, no rebound appeared in the OTCFs of solid-like nanoconfined water of *h* = 10 Å, where the OTCFs decayed monotonically as the behavior of *C*_r_(*t*) shown in Figure S3b. The HB network of solid-like nanoconfined water was broken, with less HBs formed between molecules and their neighbors, so that a molecule was easier to escape out of its HB cage, with the HBs rarely reformed after breaking. This broken HB network suggests that nanoconfined water within the nanoslit of width 10 Å had larger rotational diffusion compared with liquid-like nanoconfined waters of *h* = 15 and 20 Å.

**Figure 4:**
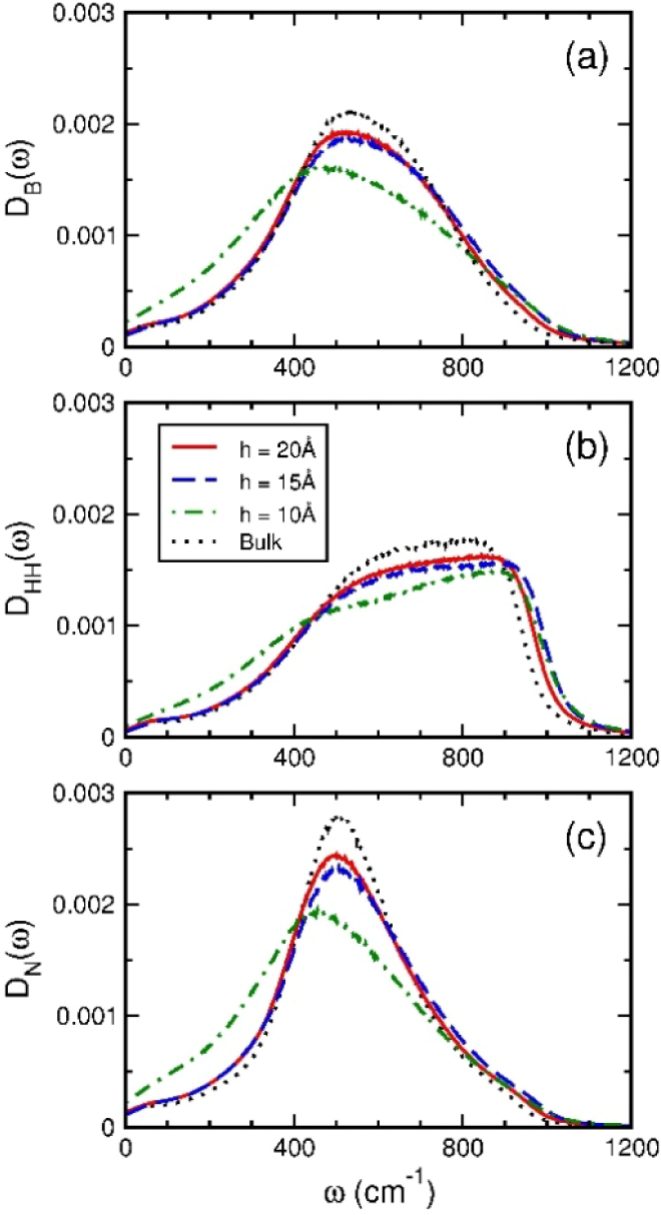
Rotational spectra of water confined within graphite nanoslits. The rotational spectra were the power spectra of molecular angular velocity autocorrelation functions generated by MD simulations.^54^ The panels are for different molecular principal axes: (a) the axis of the molecular dipole, (b) the axis parallel to the line joining two H atoms, and (c) the axis perpendicular to the molecular plane. The red solid, blue dashed, and green dot-dashed lines are for nanoslits of width 20 Å, 15 Å, and 10 Å, respectively. The black dotted lines are for TIP4P/2005 liquid water under ambient conditions.^57^

The larger rotational diffusion in solid-like nanoconfined water was indicated by the rotational spectra of nanoconfined water presented in Figure 4. The rotational spectra of liquid-like nanoconfined water were comparable to those of bulk water, whereas the rotational spectra of solid-like nanoconfined water at *h* = 10 Å shifted toward small frequencies owing to less HB number per molecule. This result is consistent with the rotational spectra of nanoconfined SPC/E waters, as shown in Figure S2b, where the nanoconfined SPC/E water within the graphite nanoslit of *h* = 10 Å was still in liquid-like states with an effective mass density somewhat lower than that of nanoconfined TIP4P/2005 water. The zero-frequency value of the rotational spectrum is related to the rotational diffusion coefficient according to the Debye theory.^67,77^ A similar low-frequency shift on the rotational spectra also occurred to molecules with anisotropic local structures in bulk water^55^ and the high-density liquid of supercooled water.^68^ As the size of the nanoslit decreased, the rotational and translational spectra of nanoconfined water were found to shift in the opposite direction.^48^ Thus, the larger rotational diffusion but lower translational diffusion of solid-like nanoconfined water suggests the decoupling between translations and rotations of water molecules confined within graphite nanoslits of size narrowed down to 10 Å, which is consistent with previous studies for water confined within nanocapillaries.^40,43,69^

The orientation trajectories for liberations of interfacial molecules within graphite nanoslits are shown in Figure S8, where the OH-group of the dangling −OH molecule swung within the water–graphite gap with fewer oscillations than the OH-group of the vertical-like molecule in connection to the next layer.

### 4.2 From Intermediate to Long Time Scales

**Figure 5:**
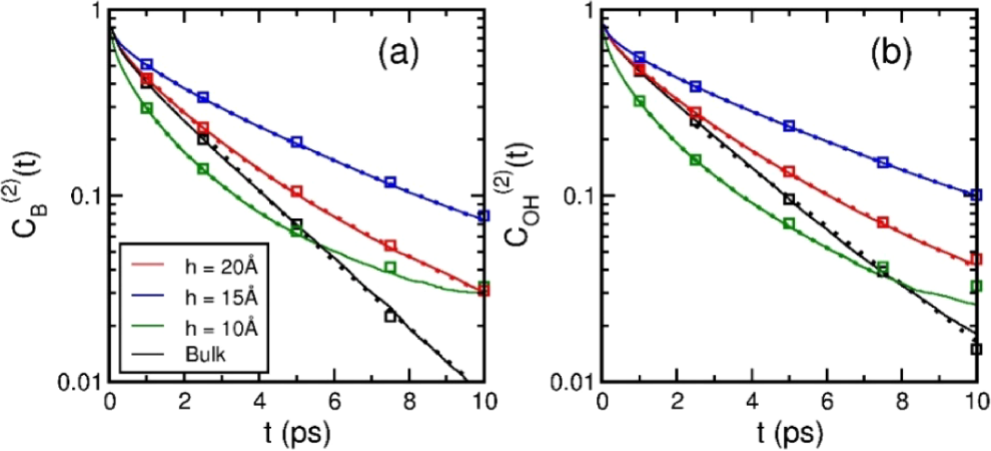
Second-rank OTCFs of nanoconfined water at intermediate time scales: (a) *C*_B_^(2)^(*t*) and (b) *C*_OH_^(2)^(*t*). The solid red, blue, and green lines are the simulation results for nanoslits of width 20 Å, 15 Å, and 10 Å, respectively, and the black lines are for TIP4P/2005 liquid water under ambient conditions. The dotted lines are the fitting by using the stretched exponential function from 1 to 10 ps for the slit widths of 20 and 15 Å, but from 1 to 5 ps and from 1 to 7.5 ps for the slit width of 10 Å in (a) and (b), respectively. The squares are the results calculated with OVHFs shown in Figure 8 according to eq 4.

**Figure 6:**
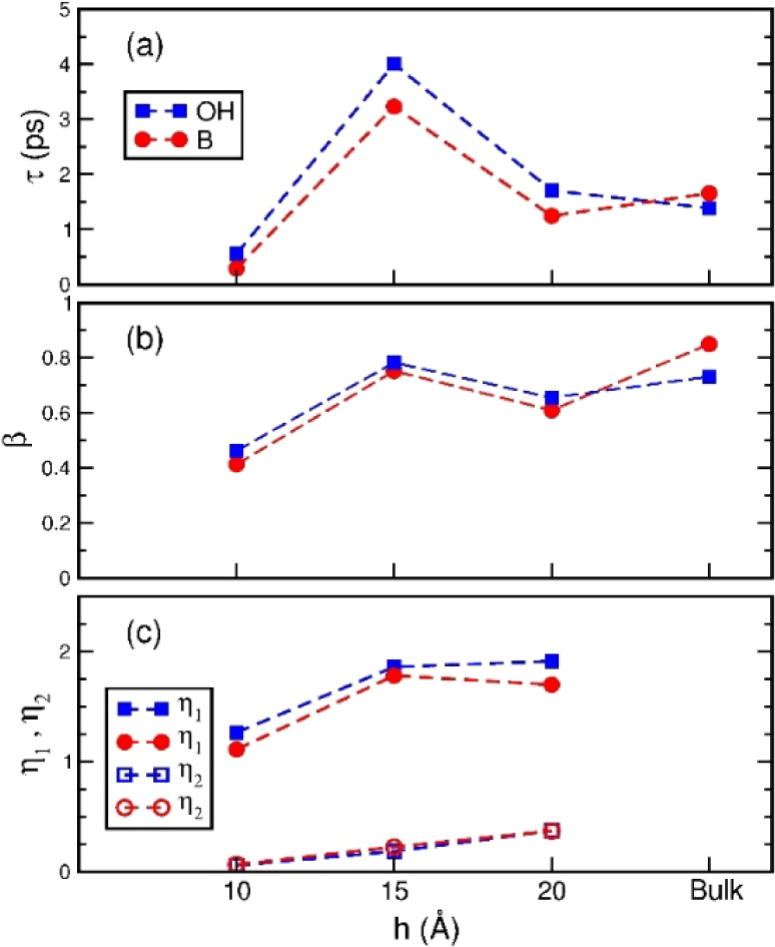
Parameters for the second-rank OTCFs of nanoconfined water at intermediate and long time scales. Panels (a) and (b) are, respectively, the relaxation time τ and the exponent β of the stretched exponential function used for fitting at intermediate time scales. In panel (c), the fill and open symbols are, respectively, the exponents η_1_ and η_2_ of power-law decays of OTCFs at different long-time regimes. Red circles and blue squares are for the OTCFs of the molecular dipole and OH-group, respectively. The dashed lines guide the eye for the variation of parameters with the slit width, with the data at the right-hand side for TIP4P/2005 bulk water under ambient conditions.

Figure 5 shows *C*_B_^(2)^(*t*) and *C*_OH_^(2)^(*t*) of nanoconfined water up to 10 ps, where the OTCFs from 1 to 10 ps, generally, were fitted by using a stretched exponential function *A* exp[−(*t*/τ)^β^)], with the values of relaxation time τ and stretched exponent β displayed in Figure 6a,b, respectively. At *h* = 20 Å, the OTCFs were slightly deviated from the exponential decay of bulk water, with τ similar to the value of bulk water and β slightly decreasing due to heterogeneous local structures within the nanoslit. At *h* = 15 Å, the deviation of the OTCFs from that of bulk water was enhanced, with τ almost twice of the bulk value, which is consistent with previous reports for purely hydrophobic nanoslits, where slow molecular reorientations were observed in the proximity of an interface.^40,43^ The β-value of *h* = 15 Å was closer to that of bulk water, possibly, due to a slight increase on the HB number of inner molecules within this nanoslit.^48^ However, at *h* = 10 Å, the OTCFs of solid-like nanoconfined water changed significantly in behavior to a concave curve in the log-normal plot, such that the time interval of the fitting by using stretched exponential function was only up to 5 and 7.5 ps for *C*_B_^(2)^(*t*) and *C*_OH_^(2)^(*t*), respectively, and the fitting values of τ and β were descent considerably from the data of *h* = 15 Å, where τ was less than 0.5 ps, possibly, due to the broken HB network, where molecules reorient more easily.

**Figure 7:**
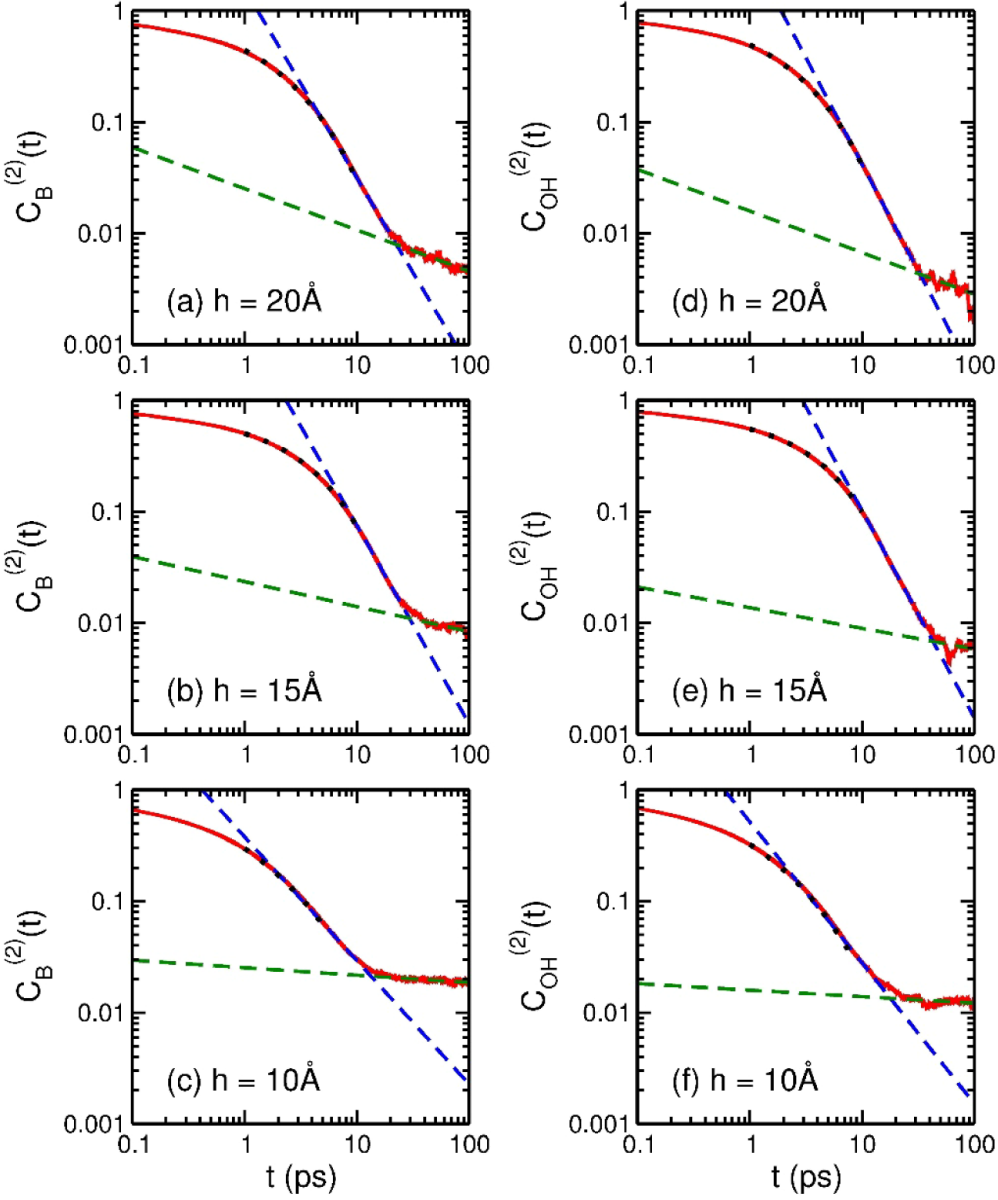
Log–log plots for *C*_B_^(2)^(*t*) and *C*_OH_^(2)^(*t*) of water confined within graphite nanoslits. The red solid lines are simulation results up to 100 ps. The black dotted lines are the fitting results by using a stretched exponential function from 1 to 10 ps in general. The blue dashed lines are the fitting by using a power-law function  at time scales somewhat overlapped with the stretched exponential decay. The green dashed lines show the fitting by using a second power law  beyond the transition time *t*_2_, which is near the crossing of blue and green dashed lines.

Figure 7 shows the log–log plots of *C*_B_^(2)^(*t*) and *C*_OH_^(2)^(*t*) of water confined within graphite nanoslits from 0.1 to 100 ps, where the time profiles of the second-rank OTCFs were described with the stretched-exponential, power-law, and power law functions at different time scales. Beyond a time scale somewhat overlapped with the stretched exponential decay, the OTCFs gradually changed to a power-law behavior , where the two behaviors merged smoothly near *t*_1_, roughly, 5, 8, and 2 ps for *h =* 20 Å, 15 Å, and 10 Å, respectively, where *t*_1_ of the three nanoslits are in accordance with the order of their relaxation times τ. As shown in Figure 6c, the η_1_ values of the three nanoconfined water were between one and two, higher than that of water confined within hydrophilic silica pores.^29,30,36,38^ But, over 10 ps, the behavior of the second-rank OTCF turned to another power law , where η_2_ had a value smaller than η_1_ and decreased almost linearly from 0.37 to 0.06 as reducing the slit size from 20 to10 Å. The second transition was observed more clearly than the first one, where the transition time *t*_2_ depends on the slit size and the molecular unit vector studied, with values estimated as 22, 30, and 14 ps for *C*_B_^(2)^(*t*) and as 33, 42, and 18 ps for *C*_OH_^(2)^(*t*) for slit widths of 20 Å, 15 Å, and 10 Å, respectively.

**Figure 8:**
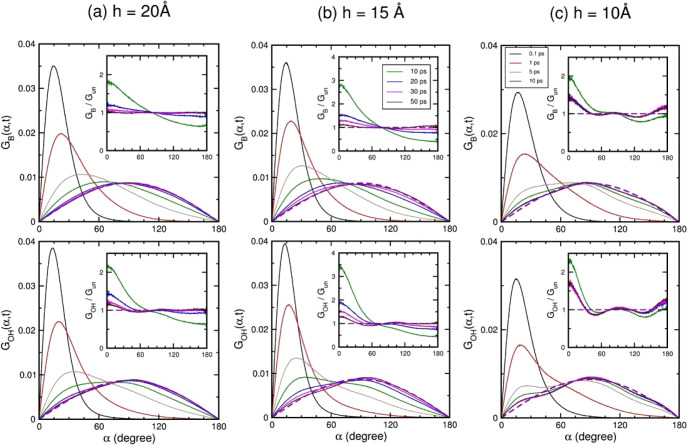
OVHF *G*_ν_(α,*t*) of molecules confined within graphite nanoslits. Columns from left to right are for nanoslits of width 20 Å, 15 Å, and 10 Å. The upper and lower panels present the OVHFs calculated with *ê*_B_(*t*) and *ê*_OH_(*t*), respectively. The OVHFs are shown at 0.1 (black), 1 (red), 5 (brown), 10 (green), 20 (blue), 30 (magenta), and 50 (maroon) ps. The violet dashed lines indicate the OVHF *G*_un_(α) of free molecules in random orientations. All OVHFs are normalized to one. The insets present the ratio of *G*_ν_(α,*t*) with respect to *G*_un_(α) at 10, 20, 30, and 50 ps.

To realize the naïve behavior of the second-rank OTCFs shown in Figure 7, we calculated OVHFs *G*_B_(α,*t*) and *G*_OH_(α,*t*) with *ê*_B_(*t*) and *ê*_OH_(*t*), respectively, for nanoconfined water within graphite nanoslits, where their time variations from 0.1 to 50 ps are presented in Figure 8. The second-rank OTCFs evaluated with the second moment of the OVHFs according to eq 4 are presented in Figure 5 at several times, and the results agreed well with *C*_B_^(2)^(*t*) and *C*_OH_^(2)^(*t*) obtained directly from MD simulations. The agreements demonstrate eq 4, which gives a relation between the second-rank OTCF and the OVHF. Hence, the second-rank OTCF can be considered an indicator for molecular reorientations of a system during a process approaching to random orientations as free molecules, with the zero of the second-rank OTCF signaling the time for achieving full randomness.

As shown in Figure 8, the OVHF of nanoconfined water at 0.1 ps displays a sharp peak at α ≈ 15°, due to the HB cage effect on molecular reorientations restricted by their neighbors, where the sharp peak is similar to the prediction of the wobbling-in-a cone model.^70^ Beyond the rebound due to liberations to time *t*_1_, where the second-rank OTCFs were described as a stretched exponential decay, the OVHFs of nanoconfined water spread out into the large-α regime, with the maximum decreasing in value, revealing the evacuation of molecules out of the HB cages formed by their neighbors. The evacuation can be executed by the extended jump model, which involves the large-angle jump reorientation and the slower frame reorientation of an intact HB pair,^42,71^ and the hopping dynamics between layers.^72^ The spreading of the OVHF during this time interval was similar to the self-part Van Hove function for translations in supercooled liquids, which can be depicted with the mode-coupling theory.^73^ Comparable to heterogeneous local structures in supercooled liquids for translational motions,^74^ the stretched exponential decay of molecular reorientation in nanoconfined water was resulted from various HB configurations of confined molecules, which are different from the major D2A2, and sharp changes in the local structure of molecules at an interfacial layer.

After the evacuation out of an HB cage, molecules lost the memory of initial orientations, and their reorientations became diffusive on a spherical surface of unit radius at time scales longer than the reorientation time of the extended jump model.^42^ Generally, *G*_ν_(α,*t*) of liquid water at orientation diffusion time scales shows a behavior quite different from the self-part Van Hove function for translational motions in a 3D space,^75^ but approaches to *G*_un_(α) of free molecules. Indicated by our results, *G*_ν_(α,*t*) of liquid-like nanoconfined water gradually approached to *G*_un_(α) with increasing time and they became almost equal at 50 ps, evidenced by their ratios shown in the insets of Figure 8. However, for solid-like nanoconfined water at 5 ps from the initial, *G*_B_(α,*t*) exhibited a shoulder at small α, and *G*_OH_(α,*t*) displayed a bimodal distribution with a peak similar in position as at short times and another peak appeared near α = 90°. This bimodal distribution indicates some molecules at 5 ps possibly residing in the HB cage formed by their neighbors. At 50 ps, both *G*_B_(α,*t*) and *G*_OH_(α,*t*) were still deviated from *G*_un_(α), where the deviations made their second moments slightly different from that of *G*_un_(α). This difference left the second-rank OTCFs a finite residue at long times according to eq 4, where a long-time residue of the second-rank OTCF was observed by experiments and simulations in the study for molecular reorientations of nanoconfined water.^26,28,29^

**Figure 9:**
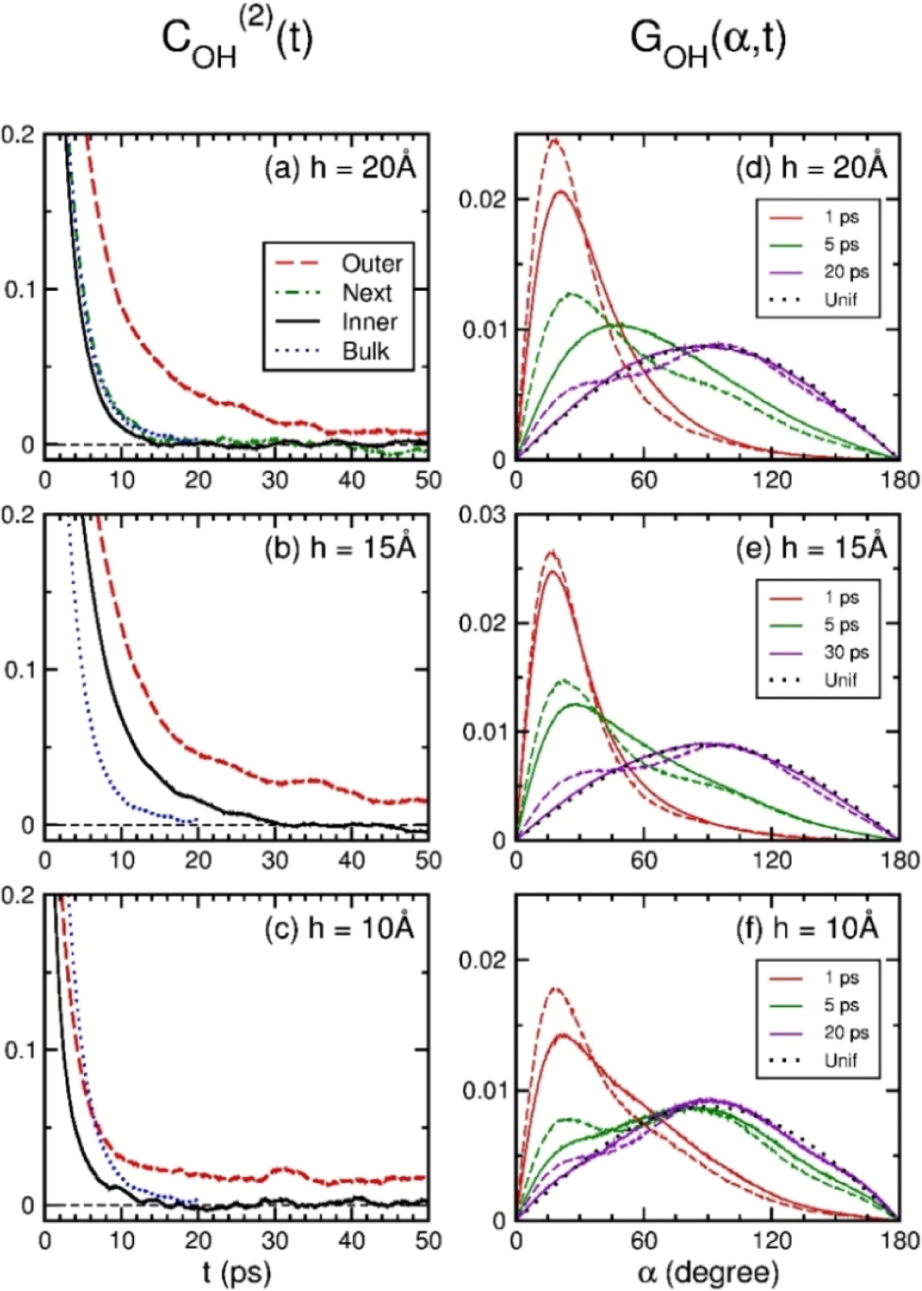
OTCF *C*_OH_^(2)^(*t*) and OVHF *G*_OH_(α,*t*) of water layers within a graphite nanoslit. The panels from top to bottom are for nanoslits of width 20 Å, 15 Å, and 10 Å, respectively. On the left column, the black-solid, green-dot-dashed, and red-dashed lines are OTCFs of inner, next, and outer layers, respectively. The blue dotted lines are for TIP4P/2005 liquid water under ambient conditions. On the right column, the solid and dashed lines are OVHFs of inner and outer layers, respectively. The red, green, and violet (magenta) lines are OVHFs at 1, 5, and 20 (30) ps, respectively. The black dotted lines are the OVHFs of free molecules in random orientations.

In our previous work,^48^ water molecules confined within a graphite nanoslit were classified into distinct layers, where the number fluctuation of a layer was about 1–2% for liquid-like nanoconfined water and less than 1% for solid-like nanoconfined water. Here, the OTCFs of a layer were calculated by averaging over molecules initially located in the layer. *C*_OH_^(2)^(*t*) of different layers within a graphite nanoslit are presented in Figure 9, and *C*_B_^(2)^(*t*) are shown in Figure S10. According to our results, for *h* = 20 Å, *C*_OH_^(2)^(*t*) of the inner and next layers decayed similarly to that of bulk water, where the HB numbers per molecule *n*_HB_ of the two layers were close to that of bulk water;^48^ near 30 ps, *C*_OH_^(2)^(*t*) of the two layers almost vanished. For *h* = 15 Å, the decay of *C*_OH_^(2)^(*t*) of the inner layer was slower than that of bulk water, with an extinction near 40 ps, where *n*_HB_ of the inner layer was near 3.74, which is higher than the bulk value. For *h* = 10 Å, *C*_OH_^(2)^(*t*) of the inner layer, with *n*_HB_ ≈ 3.434, decayed fast than that of bulk water and was extinct near 20 ps, due to the broken HB network of solid-like nanoconfined water.

Within a nanoslit, *C*_OH_^(2)^(*t*) of an outer layer generally had a value of 0.01 near 50 ps, decaying much slowly than that of the inner layer. Thus, an outer layer within a graphite nanoslit had much slow reorientation relaxation relative to the inner layer. In formulizm, the second-rank OTCF of nanoconfined water is a sum of OTCFs of all layers weighted by layer molecular fractions.^48^ The contributions of inner and outer layers gave rise to a power-law decay in *C*_OH_^(2)^(*t*) of nanoconfined water at time scales beyond the stretched exponential decay; the same situation worked for *C*_B_^(2)^(*t*). Thus, the first power-law decay of the second-rank OTCFs shown in Figure 7 was attributed to the quite distinct decay rates of reorientation relaxation between the inner and interfacial layers within a graphite nanoslit. This is consistent with the two-state description of nanoconfined water.^36,53^

Based on the aforementioned results, *C*_OH_^(2)^(*t*) of an inner layer almost vanished near the transition time *t*_2_, where the extinction of *C*_OH_^(2)^(*t*) was evidenced by the equivalence between *G*_OH_(α,*t*) and *G*_un_(α) for an inner layer shown on the right column of Figure 9. At times near *t*_2_, molecular orientations in the inner region of a nanoslit were almost equilibrium and as random as free molecules. However, it was not the case for molecular reorientations at an outer layer, which *G*_OH_(α,*t*) near *t*_2_ was still deviated from *G*_un_(α) by displaying a bimodal distribution. This deviation made *C*_OH_^(2)^(*t*) of an outer layer a residual value persisting for much longer times. Hence, the extinction on the reorientation relaxation of the inner region caused the second-rank OTCFs of nanoconfined water within a nanoslit to have a second transition, beyond which the OTCFs still decayed in the power law but with a power smaller than the first one.

Beyond the second transition, the slow decay of the second-rank OTCF of nanoconfined water resulted from the interfacial molecules, whose reorientations were not completely free even at such long times but were restricted by their interactions with the nearby confining wall of graphite. As shown in Figure S9, the restrictions were illustrated by the long-time orientation trajectories of parallel-like, vertical-like, and dangling −OH molecules initially located in an interfacial layer within a nanoslit. Up to 100 ps, the orientation trajectories of these molecules were found not to cover the unit-sphere surface uniformly. Except for the dangling OH-group initially, the orientation trajectory of a molecular unit vector showed a general feature of rarely going through the region with its polar angle less than 30°, where the unit vector points to the confining wall nearby. This is a result that interfacial molecules have preferential orientations relative to a nearby hydrophobic flat surface, indicated by the solid-angle distributions shown in Figure S4 and by other studies for water molecules near a hydrophobic surface.^56,58,76^

## 5 Conclusions

In this paper, we have used MD simulations to study the reorientation dynamics of water confined within graphite nanoslits of size less than 2 nm, where the confining walls of the nanoslit are flat surfaces without patchiness and local net charge. Subject to hydrophobic interactions from graphite, water confined within a nanoslit was generally distinguished into inner and interfacial layers parallel to the confining walls, where the average water density within the geometric space of a slit was close to that of liquid water under ambient conditions. For slit widths of 20 and 15 Å, nanoconfined water had a liquid-like local structure, which deviated from that of bulk water due to the slit confinement, where the HB network decreases in four HB molecules but increases in three and two HB molecules instead. However, within the nanoslit of width 10 Å, the local structure of nanoconfined water was solid-like and, overall, formed a broken HB network with a significant reduction in four HB molecules and more on other HB configurations, especially, bifurcated HB acceptors.

In our studies, the reorientation dynamics of nanoconfined water was described by the second-rank OTCFs of two molecular unit vectors. On short time scales, the OTCFs of liquid-like nanoconfined water displayed a rebound due to molecular liberations, similar to bulk water. However, no rebound appeared in the OTCFs of solid-like nanoconfined water due to its broken HB network, which makes molecules reorient more easily. This indicates a decoupling between parallel translations and rotations of solid-like nanoconfined water, where the decoupling was evidenced by the shifts in the opposite direction of translational and rotational vibrational spectra with respect to the corresponding spectra of bulk water as the size of the graphite nanoslit was narrowed down to 10 Å.^48^ From intermediate to long time scales, the second-rank OTCFs of nanoconfined waters were found to follow stretched-exponential, power-law, and power-law decays, which were essentially caused by the confinement of the graphite nanoslit with a size less than 2 nm. To understand this naïve behavior of reorientation relaxation, the second-rank OTCF was formulated in relation to the OVHF, with the formula evidenced numerically. Hence, the second-rank OTCF was interpreted alternatively as a measure of the deviation of the OVHF of a molecular system from that of free molecules in random orientation

Illustrated with the OVHFs at related time scales, the stretched-exponential decay of the second-rank OTCFs resulted from evacuating molecules out of the HB cages formed by their neighbors. In the following, the first power-law decay of the second-rank OTCFs was attributed to distinct relaxation rates of molecular reorientation between the inner and interfacial layers within a nanoslit. As indicated by the OVHFs of layers, as the reorientation relaxation of an inner layer vanished but that of interfacial molecules persisted, a second transition occurred to the second-rank OTCFs of nanoconfined water, which still decayed in a power law but with a power smaller than the first one. Thus, the long-time residue of the second-rank OTCF resulted from extremely slow reorientation relaxations of interfacial molecules owing to their interactions with graphite walls.

Within a graphite nanoslit, the homogeneous nanoconfined water in the direction parallel to the confining walls provides an advantage to observe the second transition in the second-rank OTCF. However, within nanopores made of other materials with interfacial roughness and impurities or other shapes in geometry, the second transition in the second-rank OTCF might be blurred or unobserved due to the disturbance of the nanopore interface, where the inner water molecules may not be so easy to get equilibrium and random in orientation as free molecules. It would be interesting to examine the naïve behavior of reorientation dynamics found within graphite nanoslits to occur to water or fluids confined within other nanopores.

## Supporting Information Available

Molecular dynamics simulations; comparisons of water models; HB dynamics; orientations of interfacial molecules; interlayer HBs; HB configurations of water layers; orientational trajectories of interfacial molecules (PDF)

## References

[ref1] Laage D.; Stirnemann G.; Sterpone F.; Rey R.; Hynes J. T. Reorientation and allied dynamics in water and aqueous solutions. Annu. Rev. Phys. Chem. 2011, 62, 395–416. 10.1146/annurev.physchem.012809.103503. 21219140

[ref2] Bagchi B. Water dynamics in the hydration layer around proteins and micelles. Chem. Rev. 2005, 105, 3197–3219. 10.1021/cr020661+. 16159150

[ref3] Ando K.; Hynes J. T. Molecular mechanism of HCl acid ionization in water: *Ab initio* potential energy surfaces and Monte Carlo simulations. J. Phys. Chem. B 1997, 101, 10464–10478. 10.1021/jp970173j.

[ref4] Marx D.; Tuckerman M. E.; Hutter J.; Parrinello M. The nature of the hydrated excess proton in water. Nature 1999, 397, 601–604. 10.1038/17579.

[ref5] Berkelbach T. C.; Lee H. S.; Tuckerman M. E. Concerted hydrogen-bond dynamics in the transport mechanism of the hydrated proton: A first-principles molecular dynamics study. Phys. Rev. Lett. 2009, 103, 238302 10.1103/PhysRevLett.103.238302. 20366181

[ref6] Agmon N. The Grotthuss mechanism. Chem. Phys. Lett. 1995, 244, 456–462. 10.1016/0009-2614(95)00905-J.

[ref7] Fecko C. J.; Loparo J. J.; Roberts S. T.; Tokmakoff A. Local hydrogen bonding dynamics and collective reorganization in water: Ultrafast infrared spectroscopy of HOD/D2O. J. Chem. Phys. 2005, 122, 054506 10.1063/1.1839179. 15740338

[ref8] Laage D.; Hynes J. T. A molecular jump mechanism of water reorientation. Science 2006, 311, 832–835. 10.1126/science.1122154. 16439623

[ref9] Laage D.; Hynes J. T. On the molecular mechanism of water reorientation. J. Phys. Chem. B 2008, 112, 14230–14242. 10.1021/jp805217u. 18942871

[ref10] Ohmine I.; Tanaka H. Fluctuation, relaxations, and hydration in liquid water: Hydrogen-bond rearrangement dynamics. Chem. Rev. 1993, 93, 2545–2566. 10.1021/cr00023a011.

[ref11] Schulz R.; von Hansen Y.; Daldrop J. O.; Kappler J.; Noé F.; Netz R. R. Collective hydrogen-bond rearrangement dynamics in liquid water. J. Chem. Phys. 2018, 149, 244504 10.1063/1.5054267. 30599706

[ref12] Offei-Danso A.; Morzan U. N.; Rodriguez A.; Hassanali A.; Jelic A. The collective burst mechanism of angular jumps in liquid water. Nat. Commun. 2023, 14, 1345 10.1038/s41467-023-37069-9. 36906703 PMC10008639

[ref13] Lawrence C. P.; Skinner J. L. Vibrational spectroscopy of HOD in liquid D2O. III. Spectral diffusion, and hydrogen-bonding and rotational dynamics. J. Chem. Phys. 2003, 118, 264–272. 10.1063/1.1525802.

[ref14] Bakker H. J.; Skinner J. L. Vibrational spectroscopy as a probe of structure and dynamics in liquid water. Chem. Rev. 2010, 110, 1498–1517. 10.1021/cr9001879. 19916491

[ref15] Fayer M. D.; Levinger N. E. Analysis of water in confined geometries and at interfaces. Annu. Rev. Anal. Chem. 2010, 3, 89–107. 10.1146/annurev-anchem-070109-103410. 20636035

[ref16] Giovambattista N.; Rossky P. J.; Debenedetti P. G. Computational studies of pressure, temperature, and surface effects on the structure and thermodynamics of confined water. Annu. Rev. Phys. Chem. 2012, 63, 179–200. 10.1146/annurev-physchem-032811-112007. 22475337

[ref17] Giovambattista N.; Rossky P. J.; Debenedetti P. G. Effect of temperature on the structure and phase behavior of water confined by hydrophobic, hydrophilic, and heterogeneous surfaces. J. Phys. Chem. B 2009, 113, 13723–13734. 10.1021/jp9018266. 19435300

[ref18] Lin L.; Ge Y.; Zhang H.; Wang M.; Xiao D.; Ma D. Heterogeneous catalysis in water. JACS Au 2021, 1, 1834–1848. 10.1021/jacsau.1c00319. 34841403 PMC8611672

[ref19] Lynch C. I.; Rao S.; Sansom M. S. P. Water in nanopores and biological channels: A molecular simulation perspective. Chem. Rev. 2020, 120, 10298–10335. 10.1021/acs.chemrev.9b00830. 32841020 PMC7517714

[ref20] Wei Y.; Zhang Y.; Gao X.; Ma Z.; Wang X.; Gao C. Multilayered graphene oxide membranes for water treatment: A review. Carbon 2018, 139, 964–981. 10.1016/j.carbon.2018.07.040.

[ref21] Giri A. K.; Teixeira F.; Cordeiro M. N. D. S. Salt separation from water using graphene oxide nanochannels: A molecular dynamics simulation study. Desalination 2019, 460, 1–14. 10.1016/j.desal.2019.02.014.

[ref22] Hu R.; He Y.; Zhang C.; Zhang R.; Li J.; Zhu H. Graphene oxide-embedded polyamide nanofiltration membranes for selective ion separation. J. Mater. Chem. A 2017, 5, 25632–25640. 10.1039/C7TA08635K.

[ref23] Muñoz-Santiburcio D.; Marx D. Confinement-controlled aqueous chemistry within nanometric slit pores. Chem. Rev. 2021, 121, 6293–6320. 10.1021/acs.chemrev.0c01292. 34006106

[ref24] Tan H. -S.; Piletic I. R.; Fayer M. D. Orientational dynamics of water confined on a nanometer length scale in reverse micelles. J. Chem. Phys. 2005, 122, 174501 10.1063/1.1883605. 15910039

[ref25] Piletic I. R.; Moilanen D. E.; Spry D. B.; Levinger N. E.; Fayer M. D. Testing the core/shell model of nanoconfined water in reverse micelles using linear and nonlinear IR spectroscopy. J. Phys. Chem. A 2006, 110, 4985–4999. 10.1021/jp061065c. 16610816

[ref26] Pieniazek P. A.; Lin Y. S.; Chowdhary J.; Ladanyi B. M.; Skinner J. L. Vibrational spectroscopy and dynamics of water confined inside reverse micelles. J. Phys. Chem. B 2009, 113, 15017–15028. 10.1021/jp906784t. 19842648

[ref27] Levinger N. E.; Swafford L. A. Ultrafast dynamics in reverse micelles. Annu. Rev. Phys. Chem. 2009, 60, 385–406. 10.1146/annurev.physchem.040808.090438. 18999990

[ref28] Martinez A. V.; Dominguez L.; Małolepsza E.; Moser A.; Ziegler Z.; Straub J. E. Probing the structure and dynamics of confined water in AOT reverse micelles. J. Phys. Chem. B 2013, 117, 7345–7351. 10.1021/jp402270e. 23687916 PMC3709849

[ref29] Scodinu A.; Fourkas J. T. Comparison of the orientational dynamics of water confined in hydrophobic and hydrophilic nanopores. J. Phys. Chem. B 2002, 106, 10292–10295. 10.1021/jp026349l.

[ref30] Farrer R. A.; Fourkas J. T. Orientational dynamics of liquids confined in nanoporous sol-gel glasses studied by optical Kerr effect spectroscopy. Acc. Chem. Res. 2003, 36, 605–612. 10.1021/ar0200302. 12924957

[ref31] Faraone A.; Liu L.; Mou C. Y.; Shih P. C.; Copley J. R. D.; Chen S. H. Translational and rotational dynamics of water in mesoporous silica materials: MCM-41-S and MCM-48-S. J. Chem. Phys. 2003, 119, 3963–3971. 10.1063/1.1584653.

[ref32] Zanotti J. M.; Bellissent-Funel M. C.; Chen S. H. Relaxational dynamics of supercooled water in porous glass. Phys. Rev. E 1999, 59, 3084–3093. 10.1103/PhysRevE.59.3084.

[ref33] Taschin A.; Bartolini P.; Marcelli A.; Righini R.; Torre R. Supercooling and freezing processes in nanoconfined water by time-resolved optical Kerr effect spectroscopy. J. Phys.: Condens. Matter 2015, 27, 194107 10.1088/0953-8984/27/19/194107. 25924077

[ref34] Plenum. Reverse Micelles: Biological and Technological Relevance of Amphiphilic. In Structures in Apolar Media, Luisi P. L.; Straub B. E., Eds.; Plenum: New York, 1984.

[ref35] Brinker C. J.; Scherer G. W. Sol-Gel Science: The Physics and Chemistry of Sol-Gel Processing; Academic Press: New York, 1990.

[ref36] Laage D.; Thompson W. H. Reorientation dynamics of nanoconfined water: Power-law decay, hydrogen-bond jumps, and test of a two-state model. J. Chem. Phys. 2012, 136, 044513 10.1063/1.3679404. 22299897

[ref37] Thompson W. H. Perspective: Dynamics of confined liquids. J. Chem. Phys. 2018, 149, 170901 10.1063/1.5057759. 30408973

[ref38] Fogarty A. C.; Duboué-Dijon E.; Laage D.; Thompson W. H. Origins of the non-exponential reorientation dynamics of nanoconfined water. J. Chem. Phys. 2014, 141, 18C523 10.1063/1.4896983. 25399188

[ref39] Milischuk A. A.; Ladanyi B. M. Structure and dynamics of water confined in silica nanopores. J. Chem. Phys. 2011, 135, 174709 10.1063/1.3657408. 22070319

[ref40] Romero-Vargas Castrillón S.; Giovambattista N.; Aksay L. A.; Debenedetti P. G. Effect of surface polarity on the structure and dynamics of water in nanoscale confinement. J. Phys. Chem. B 2009, 113, 1438–1446. 10.1021/jp809032n. 19143545

[ref41] Stirnemann G.; Rossky P. J.; Hynes J. T.; Laage D. Water reorientation, hydrogen-bond dynamics and 2D-IR spectroscopy next to an extended hydrophobic surface. Faraday Discuss. 2010, 146, 263–281. 10.1039/b925673c. 21043427

[ref42] Stirnemann G.; Castrillón S. R. V.; Hynes J. T.; Rossky P. J.; Debenedetti P. G.; Laage D. Non-monotonic dependence of water reorientation dynamics on surface hydrophilicity: Competing effects of the hydration structure and hydrogen-bond strength. Phys. Chem. Chem. Phys. 2011, 13, 19911–19917. 10.1039/c1cp21916b. 21897944

[ref43] Romero-Vargas Castrillón S.; Giovambattista N.; Aksay I. A.; Debenedetti P. G. Evolution from surface-influenced to bulk-like dynamics in nanoscopically confined water. J. Phys. Chem. B 2009, 113, 7973–7976. 10.1021/jp9025392. 19449830

[ref44] Radha B.; Esfandiar A.; Wang F. C.; Rooney A. P.; Gopinadhan K.; Keerthi A.; Mishchenko A.; Janardanan A.; Blake P.; Fumagalli L.; Lozada-Hidalgo M.; Garaj S.; Haigh S. J.; Grigorieva I. V.; Wu H. A.; Geim A. K. Molecular transport through capillaries made with atomic-scale precision. Nature 2016, 538, 222–225. 10.1038/nature19363. 27602512

[ref45] Neek-Amal M.; Peeters F. M.; Grigorieva I. V.; Geim A. K. Commensurability effects in viscosity of nanoconfined water. ACS Nano 2016, 10, 3685–3692. 10.1021/acsnano.6b00187. 26882095

[ref46] Palchowdhury S.; Mukherjee K.; Maroncelli M. What do far-infrared spectra of solitary water in “water-in-solvent” systems reveal about water’s solvation and dynamics?. J. Chem. Phys. 2023, 159, 034502 10.1063/5.0156511. 37462284

[ref47] Van Hove L. Correlations in space and time and born approximation scattering in systems of interacting particles. Phys. Rev. 1954, 95, 249–262. 10.1103/PhysRev.95.249.

[ref48] Kuo Y. W.; Wang C. W.; Tang P. H.; Wu T. M. Layer structure and intermolecular vibrations of water confined within graphite nanoslits. Chem. Phys. Lett. 2023, 825, 140612 10.1016/j.cplett.2023.140612.

[ref49] Ruiz-Barragan S.; Forbert H.; Marx D. Anisotropic pressure effects on nanoconfined water within narrow graphene slit pores. Phys. Chem. Chem. Phys. 2023, 25, 28119–28129. 10.1039/D3CP01687K. 37818616

[ref50] Abascal J. L. F.; Vega C. A general purpose model for the condensed phases of water: TIP4P/2005. J. Chem. Phys. 2005, 123, 234505 10.1063/1.2121687. 16392929

[ref51] Han S.; Choi M. Y.; Kumar P.; Stanley H. E. Phase transitions in confined water nanofilms. Nat. Phys. 2010, 6, 685–689. 10.1038/nphys1708.

[ref52] Luzar A.; Chandler D. Hydrogen-bond kinetics in liquid water. Nature 1996, 379, 55–57. 10.1038/379055a0.

[ref53] Cicero G.; Grossman J. C.; Schwegler E.; Gygi F.; Galli G. Water confined in nanotubes and between graphene sheets: A first principle study. J. Am. Chem. Soc. 2008, 130, 1871–1878. 10.1021/ja074418+. 18211065

[ref54] Lin S. R.; Tang P. H.; Wu T. M. Local structural effects on orientational relaxation of OH-bond in liquid water over short to intermediate timescales. J. Chem. Phys. 2014, 141, 214505 10.1063/1.4902372. 25481150

[ref55] Chang S. L.; Wu T. M.; Mou C. Y. Instantaneous normal mode analysis of orientational motions in liquid water: Local structural effects. J. Chem. Phys. 2004, 121, 3605–3612. 10.1063/1.1772759. 15303927

[ref56] Ruiz-Barragan S.; Muñoz-Santiburcio D.; Marx D. Nanoconfined water within graphene slit pores adopts distinct confinement-dependent regimes. J. Phys. Chem. Lett. 2019, 10, 329–334. 10.1021/acs.jpclett.8b03530. 30571135

[ref57] Tang P. H.; Fan Y. Y.; Hsu W. L.; Wu T. M. Reorientation of OH-group connecting bifurcated H-bond acceptors in liquid water. Chem. Phys. Lett. 2018, 710, 168–174. 10.1016/j.cplett.2018.08.065.

[ref58] Maurya M.; Metya A. K.; Singh J. K.; Saito S. Effects of interfaces on structure and dynamics of water droplets on a graphene surface: A molecular dynamics study. J. Chem. Phys. 2021, 154, 164704 10.1063/5.0046817. 33940844

[ref59] Abbaspour M.; Akbarzadeh H.; Salemi S.; Jalalitalab E. Density-dependent phase transition in nano-confinement water using molecular dynamics simulation. J. Mol. Liq. 2018, 250, 26–34. 10.1016/j.molliq.2017.11.162.

[ref60] Dix J.; Lue L.; Carbone P. Why different water models predict different structures under 2D confinement. J. Comput. Chem. 2018, 39, 2051–2059. 10.1002/jcc.25369. 30226923

[ref61] Woutersen S.; Emmerichs U.; Bakker H. J. Femtosecond Mid-IR pump-probe spectroscopy of liquid water: Evidence for a two-component structure. Science 1997, 278, 658–660. 10.1126/science.278.5338.658.

[ref62] Ludwig R.; Weinhold F.; Farrar T. C. Experimental and theoretical determination of the temperature dependence of deuteron and oxygen quadrupole coupling constants of liquid water. J. Chem. Phys. 1995, 103, 6941–6950. 10.1063/1.470371.

[ref63] Ropp J.; Lawrence C.; Farrar T. C.; Skinner J. L. Rotational motion in liquid water is anisotropic: A nuclear magnetic resonance and molecular dynamics simulation study. J. Am. Chem. Soc. 2001, 123, 8047–8052. 10.1021/ja010312h. 11506561

[ref64] Berne B. J.; Pecora R. Dynamic Light Scattering; John Whiley: New York, 1976.

[ref65] Reichl L. E. A Modern Course in Statistical Physics, 2nd ed.; John Wiley: New York, 1998.

[ref66] Lynden-Bell R. M.; Steele W. A. A model for strongly hindered molecular reorientation in liquids. J. Phys. Chem. 1984, 88, 6514–6518. 10.1021/j150670a013.

[ref67] Debye P. Polar Molecules; The Chemical Catalog Company: New York, 1929.

[ref68] Tang P. H.; Wu T. M. Instantaneous normal mode analysis for OKE reduced spectra of liquid and supercooled water: Contributions of low-density and high-density liquids. J. Mol. Liq. 2020, 301, 112363 10.1016/j.molliq.2019.112363.

[ref69] Harpham M. R.; Ladanyi B. M.; Levinger N. E.; Herwig K. W. Water motion in reverse micelles studied by quasielastic neutron scattering and molecular dynamics simulations. J. Chem. Phys. 2004, 121, 7855–7868. 10.1063/1.1792592. 15485248

[ref70] Lipari G.; Szabo A. Effect of liberational motion on fluorescence depolarization and nuclear magnetic resonance relaxation in macromolecules and membranes. Biophys. J. 1980, 30, 489–506. 10.1016/S0006-3495(80)85109-5. 7260284 PMC1328752

[ref71] Piskulich Z. A.; Laage D.; Thompson W. H. Activation energies and the extended jump model: How temperature affects reorientation and hydrogen-bond exchange dynamics in water. J. Chem. Phys. 2020, 153, 074110 10.1063/5.0020015. 32828097

[ref72] Qiao Z.; Xie W. J.; Cai X.; Gao Y. Q. Interlayer hopping dynamics of bilayer water confined between graphene sheets. Chem. Phys. Lett. 2019, 722, 153–159. 10.1016/j.cplett.2019.02.046.

[ref73] Kob K.; Andersen H. C. Testing made-coupling theory for a supercooled binary Lennard-Jones mixture: The van Hove correlation function. Phys. Rev. E 1995, 51, 4626–4641. 10.1103/PhysRevE.51.4626. 9963176

[ref74] Ediger M. D. Spatially heterogeneous dynamics in supercooled liquids. Annu. Rev. Phys. Chem. 2000, 51, 99–128. 10.1146/annurev.physchem.51.1.99. 11031277

[ref75] Hansen J. P.; McDonald I. R. Theory of Simple Liquids; Academic Press: New York, 2006.

[ref76] Giovambattista N.; Debenedetti P. G.; Rossky P. J. Effect of surface polarity on water contact angle and interfacial hydration structure. J. Phys. Chem. B 2007, 111, 9581–9587. 10.1021/jp071957s. 17658789

[ref77] Hsu C. C.; Chiu H. J.; Wang C. W.; Wu T. M. Orientation time correlation functions up to the fourth cumulant for liquid water. J. Mol. Liq. 2024, 410, 125575 10.1016/j.molliq.2024.125575.

