# Supplementary material for: Confinement Effects on Reorientation Dynamics of Water Confined within Graphite Nanoslits
Source: J Phys Chem B. 2024 Sep 23;128(39):9525–35. doi: 10.1021/acs.jpcb.4c03898 (PMC11457136; doi:10.1021/acs.jpcb.4c03898)
Supplement: Supplementary file 1 — jp4c03898_si_001.pdf [file jp4c03898_si_001.pdf]

Supporting Information

Confinement Effects on Reorientation Dynamics of Water Confined within Graphite Nanoslits

(Date: Sept. 4, 2024)

Chi-Wei Wang, Yu-Wei Kuo, Jing-Rong Zeng, Ping-Han Tang, Ten-Ming Wu[#]

Institute of Physics, National Yang Ming Chiao-Tung University,
Hsinchu 300, Taiwan

[#] Corresponding author E-mail: tmw@nycu.edu.tw

MD simulations and layer structure of nanoconfined water

In our studies, a graphite nanoslit consists of two parallel C-atom plates in AA-stacking on each side of the slit, where the distance between two inner plates is the slit geometric width h, with the details described in Ref. 1. The C-atom plates are subject to periodic boundary conditions in the x-and y-directions. Water molecules of number *N* = 1408, 1080, and 720 were simulated within nanoslits of width h = 20Å, 15Å, and 10Å, respectively, such that water mass density ρ_{geo} within a geometric slit space was near 1.015 ~ 1.038 g/cm^3, which is close to that of liquid water at ambient conditions. However, repelled by the hydrophobic interactions from C-atom plates, water molecules within a nanoslit were constrained within an effective slit width h_{eff}, which was reduced about 3.22Å from h for each nanoslit, so that the effective mass density ρ_{eff} within the constrained region was nearly 1.21, 1.32, and 1.53 g/cm^3 for h = 20Å, 15Å, and 10Å, respectively.

By using the package LAMMPS,[2] MD simulations of nanoconfined water were performed in two steps: First, bulk water of *N* molecules at 300 K was simulated in a box slightly smaller in size than the geometric slit space. After equilibrated, the final configuration of bulk water was inputted into a graphite nanoslit as the initial positions. Then, water molecules were simulated with extra Lennard-Jones (LJ) interactions of O-C atomic pairs in *NVT*-ensemble at 300 K with a time step of 1 fs for 10 ns, and continued in the *NVE*-simulation for 1 ns to collect data for our studies. In our model, the TIP4P/2005 rigid water model was used.[3] The LJ parameters of O-O and C-C pairs are taken from Ref. 3 and 4, respectively; the Lorentz-Berthelot mixing rules were applied for the LJ parameters of O-C atomic pair,[5] where their data are given in Ref. 1. All LJ potentials were truncated at 12Å and the Coulombic interactions longer than this distance were calculated with the particle-particle and particle-mesh method.[6] All *NVT*-simulations were controlled by the Nose-Hoover thermostat.[7,8]

Side views of nanoconfined water obtained from our simulations are presented in Fig. S1. Water molecules confined within a nanoslit formed layers parallel to the C-atom plates, where the layer boundaries were determined at the minima of O-atom z-density profile shown in Fig. 2 of Ref. 1. Within the nanoslit of width $h = 20$ Å, there are six layers, referred as outer, next, and inner layers in distance from a nearby C-atom plate. The nanoslits of width 15 and 10 Å involved four and three layers, respectively, where the layers were distinguished into outer and inner layers, with the inner layer occupied in the slit central region. Based on their effective mass densities, nanoconfined water was in liquid-like states within the nanoslits of width 15 Å and 20Å but in solid-like states within the nanoslit of width 10Å.

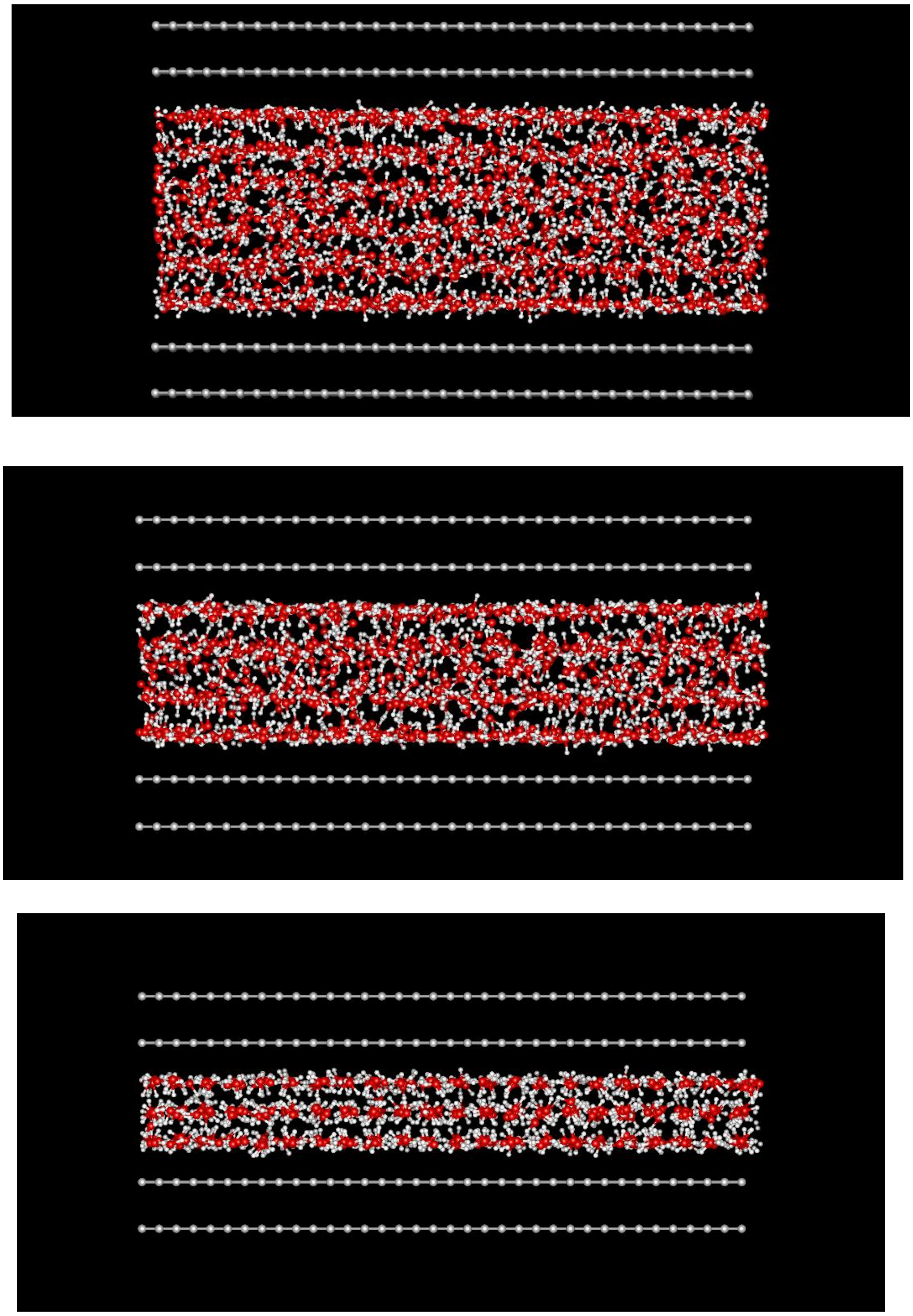

(a) (b) (c)

Fig. S1 Snapshots for side-view of water molecules confined within a graphite nanoslit of width: (a) $h = 20$ Å, (b) $h = 15$ Å, and (c) $h = 10$ Å. In each plot, the gray lines with dots are the top and bottom C-atom plates of a nanoslit. The slit width h is the distance between the two inner C-atom plates. The red and white circles indicate oxygen and hydrogen atoms of water molecule. The configurations were generated from our *NVT*-simulations.

● Comparison between TIP4P/2005 and SPC/E water models

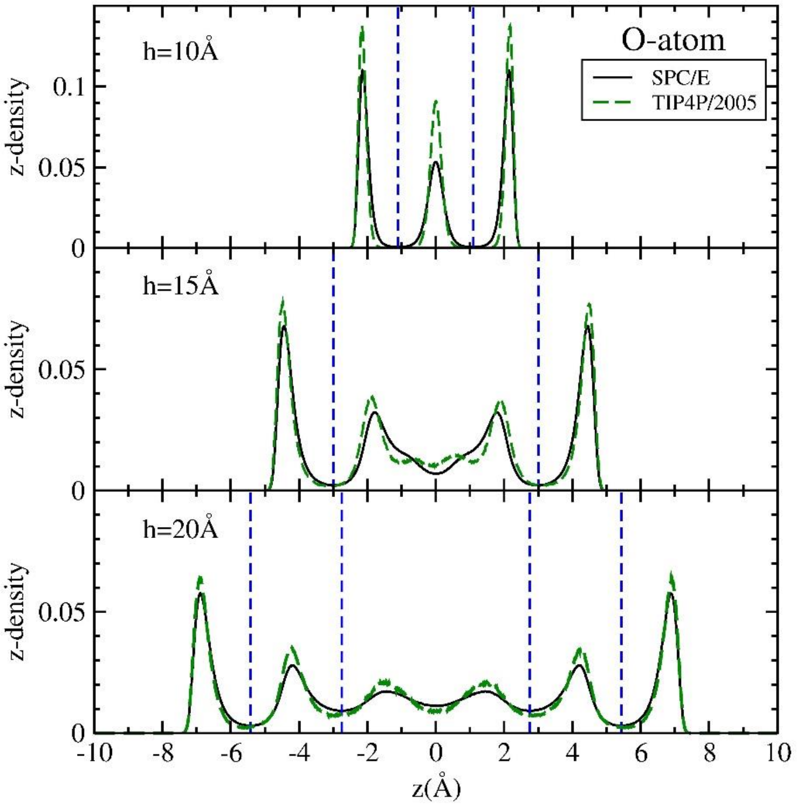

Fig. S2a Comparisons of O-atom Z-density profile calculated with TIP4P/2005 (green dash lines) and SPC/E (black solid lines) water molecules at 300 K confined within graphite nanoslits. Panels from top to bottom are for nanoslits of width $h = 10$ Å, 15 Å, and 20 Å, respectively. The geometric densities of nanoconfined water within each nanoslit were 0.999 and 1.03 g/cm^3 for SPC/E and TIP4P/2005 water models, respectively, which were close to bulk water at ambient conditions. For SPC/E model, 692, 1040, and 1386 molecules were confined within nanoslits of $h = 10$ Å, 15 Å, and 20 Å, with effective mass densities 1.47, 1.27, and 1.19 g/cm^3, respectively. The molecule number and effective mass density of TIP4P/2005 nanoconfined water within each nanoslit are given in the text. For TIP4P/2005 model, the O-atom Z-density profiles of layer are sharper in peak and, in the central region of the nanoslit of width 15Å, the profile displayed small fluctuations

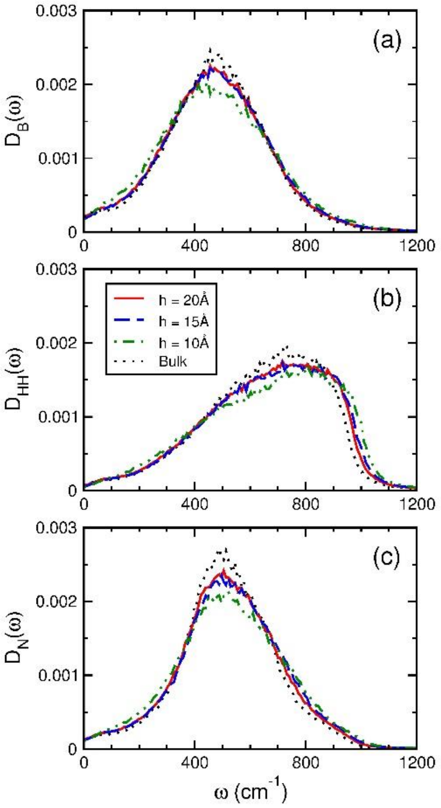

Fig. S2b Rotational spectra of SPC/E water molecules confined within graphite nanoslits. The rotational spectra were obtained by the power spectra of molecular angular velocity autocorrelation functions generated by MD simulations with similar procedures described in the text. The panels are for the axis of molecular dipole (a), the axis parallel to the line joining two H-atoms (b), and the axis perpendicular to molecular plane (c). The red solid, blue dash, and green dot-dash lines are for nanoslits of width $20Å, 15Å, \text{and } 10Å$, respectively. The black dot lines are for SPC/E bulk water at ambient conditions.[10] The rotational spectra of the three nanoconfined waters are similar as that of SPC/E bulk water. In distinction from the results of TIP4P/2005 model presented in the text, the spectra of nanoslit $h = 10$ Å has only a minor shifting toward lower frequencies, indicating that the SPC/E nanoconfined water was still in liquid-like states due to an effective mass density somewhat lower than that of TIP4P/2005 nanoconfined water.

● Time correlation functions for HB-dynamics

On HB-dynamics, the fluctuation of a HB between a molecular pair in a molecular liquid can be measured by the time correlation function[11]

$$C_x(t) = \frac{\langle h_x(t)h_x(0)\rangle}{\langle h_x\rangle},$$

where $h_x(t) = 1$ if the molecular pair is intact with a HB at time *t* and $h_x(t) = 0$ otherwise. There are two possible cases for a HB that was intact at initial:[12] In case I, the HB keeps intact up to time *t* without breaking, where $h_x(t') = 1$ for all t' smaller than t. Then, $C_x(t)$, with x = s, gives the surviving probability of a HB as a function of time. In case II, the HB of a molecular pair may break and reform many times up to time *t,* with the pair intact a HB at time *t*, where $h_x(t) = 1$ but $h_x(t')$ may be zero or one for t' smaller than t. Then, $C_x(t)$, with x = *r*, indicates the probability for a molecular pair intact a HB at time *t*, during which the HB may break and reform.

Shown in Figure S3 are $C_s(t)$ and $C_r(t)$ characterizing HB-dynamics in nanoconfined water within graphite nanoslits and in TIP4P/2005 bulk water. Both $C_s(t)$ and $C_r(t)$ of nanoconfined water decay fast than the corresponding correlation function of bulk water, where the decay time of each correlation function decreased with the nanoslit size. The lifetime τ_{HB} of a HB was estimated by the time at $C_s(\tau_{HB}) = 0.5$, where the τ_{HB} of nanoconfined water was roughly $20{\sim}10$ fs, which was much shorter than $\tau_{HB} \approx 100$ fs for bulk water. A hump appeared near 50 fs in $C_r(t)$ of nanoconfined water of $h = 20$Å and 15Å and in $C_r(t)$ of bulk water, where the hump indicates the reformation of a HB after breaking. However, no hump was observed in $C_r(t)$ of nanoconfined water of $\text{h} = 10$Å.

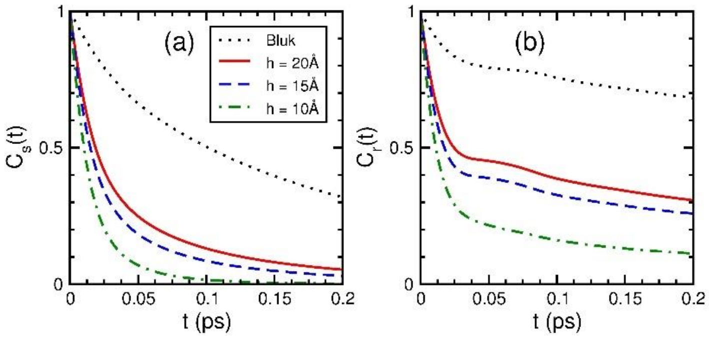

Figure S3 Time correlation function $C_s(t)$ (a) and $C_r(t)$ (b) of HB-dynamics in nanoconfined water within graphite nanoslits. The red solid, blue dash, and green dot-dash lines are for nanoslits of width 20Å, 15Å, and 10Å, respectively. The black dot lines are for TIP4P/2005 liquid water at ambient conditions.

Orientation of Interfacial Molecule

Relative to a nearby C-atom plate, specified by a unit normal vector $\hat{n}$ pointing toward the slit space as shown in an insert of Fig. S4a, the orientations of interfacial molecules can be described by two solid-angle distributions $P_N(\theta)$ and $P_{OH}(\theta)$ calculated with unit vector $\hat{e}_N$ normal to molecular plane and $\hat{e}_{OH}$ along one OH-group of water molecule, respectively, as shown in another insert of Fig. S4a. The solid-angle distribution $P_\nu(\theta)$, with $\nu = N$ and OH, is defined as

$$P_\nu(\theta) = \langle \frac{1}{2\pi \sin\theta N_l} \sum_{i=1}^{N_l} \delta(\theta - \theta_{\nu,i}) \rangle,$$

where $\theta_{\nu,i} = \cos^{-1}(\hat{e}_{\nu,i} \cdot \hat{n})$ is the angle between $\hat{e}_{\nu,i}$ of interfacial molecule i and the $\hat{n}$-vector of the nearby plate, N_l is the number of interfacial molecules, and the angular brackets denote an ensemble average. With this definition, $P_\nu(\theta) 2\pi \sin\theta d\theta$ is the probability for finding an interfacial molecule with its molecular unit vector $\hat{e}_\nu$ lying within a solid angle sustained by a right circular cone with an axis normal to the plate and an apex angle between θ and $\theta + d\theta$.

$P_N(\theta)$ and $P_{OH}(\theta)$ of water molecules confined within graphite nanoslits are shown in Fig. S 4a and 4b, respectively, where the flat dash line in each figure indicates the corresponding distribution of free molecules, which are completely random in orientation relative to the nearby plate. The solid-angle distributions have common features for nanoslits of width 15Å and 20Å. In Fig. S4a, $P_N(\theta)$ with $\theta \leq 35^\circ$ has values higher than the flat distribution of free water molecules, suggesting that the interfacial molecules with $\theta_N \leq 35^\circ$ lay almost flatly within the interfacial layer, where θ_N is the angle between $\hat{e}_N$ of a molecule and the $\hat{n}$-vector, and they were referred as parallel-like molecules. On the other hand, $P_{OH}(\theta)$ displays two regions with values above the flat distribution of free molecules, where the results are similar as for water molecules near a hydrophobic surface.[9] In the regime with $0^\circ \leq \theta \leq 30^\circ$, the interfacial molecules have one OH-bond generally pointing away from the C-atom plate and they were referred as vertical-like molecules. The second region of $P_{OH}(\theta)$, with $80 < \theta < 120^\circ$, corresponds to the second OH-group of vertical-like molecules due to the bond angle of a water molecule and the OH-groups of parallel-like molecules. In another extreme $150^\circ < \theta_{OH} < 180^\circ$, where $P_{OH}(\theta)$ has values considerably smaller than the flat distribution, the interfacial molecules had a dangling OH-group pointing to the C-atom plate and were, thus, referred as dangling-OH molecules. The orientations of the three kinds of interfacial molecule are shown in the insert of Fig. S4b.

For nanoconfined water within the nanoslit of width 10Å, $P_N(\theta)$ has a similar behavior as that of liquid-like nanoconfined water; however, the behavior of $P_{OH}(\theta)$

changes substantially at angles near 0°, with a maximum appearing near 25° rather than at 0°. This behavior change was attributed to the squeeze on an interfacial layer by the confinement, which causes the interfacial layer to have a narrow width and a much higher density than the inner layer, so that the OH-group of a vertical-like molecule in an interfacial layer was forced to deviate substantially from the direction normal to the nearby C-atom plate.

The fractions of the three kinds of interfacial molecule can be obtained by an integration over the related portion of a solid-angle distribution. For liquid-like nanoconfined water, near 45 percent of interfacial molecules were parallel-like and 27~28 percent were vertical-like; however, the dangling-OH molecules were rare, only about one to two percent. For solid-like nanoconfined water, the fractions of parallel-like and vertical-like molecules were reduced to about 40 and 18~17 percents, resepctively, but no change was found on the fraction of dangling-OH molecule.

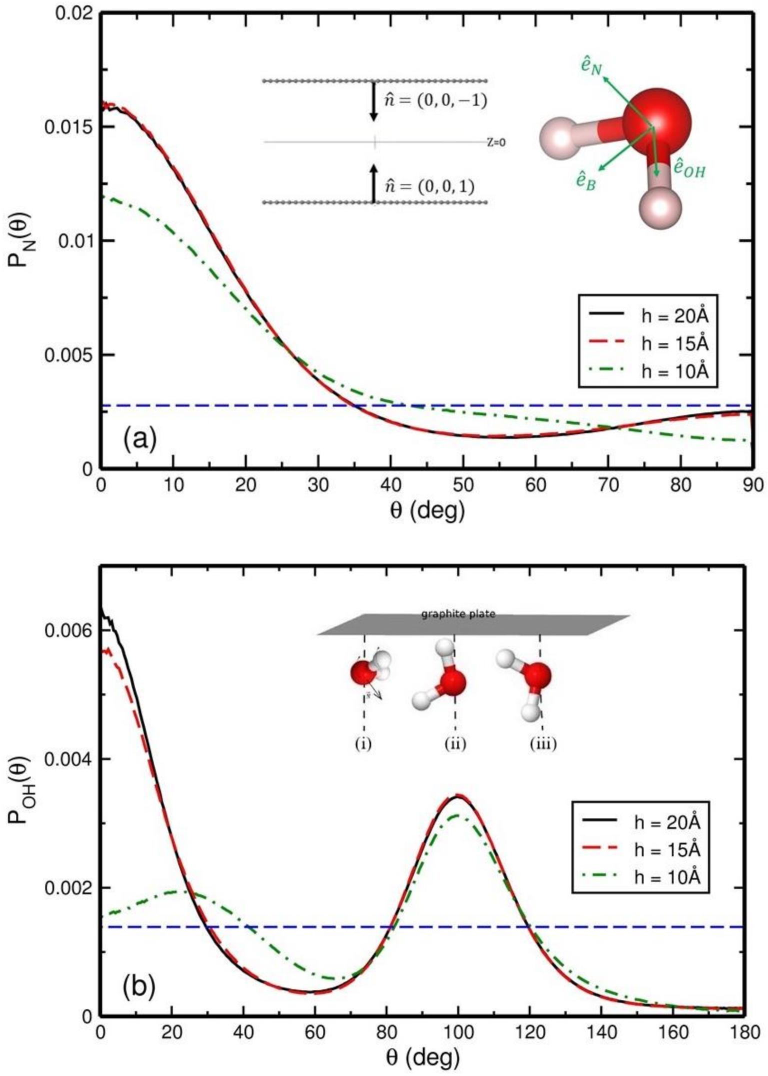

Fig. S4 Solid-angle distributions of interfacial-molecule orientation relative to a nearby C-atom plate: (a) $P_N(\theta)$ and (b) $P_{OH}(\theta)$ evaluated with $\hat{e}_N$ and $\hat{e}_{OH}$ of a molecule, respectively. The black solid, red dash, and green dot-dash lines are for nanoslits of width 20Å, 15Å, and 10Å, respectively. The blue short-dash lines indicate the distributions of free molecules in random orientations relative to the C-atom plate. In (a), the right insert indicates unit-vectors of a water molecule: $\hat{e}_B$ is a vector bisecting the HOH bond angle, $\hat{e}_N$ is normal to molecular plane, and $\hat{e}_{OH}$ is along one OH-group. The left insert specifies the $\hat{n}$-vector normal to C-atom plate of a nanoslit, pointing down ($\hat{n}$ = (0, 0, -1)) for the top plate (z > 0) and pointing up ($\hat{n}$ = (0, 0, 1)) for the bottom plate (z < 0). In (b), the insert illustrates three kinds of interfacial molecule relative to a plate: (i) parallel-like, (ii) dangling-OH, and (iii) vertical-like.

- **Interlayer HBs between interfacial and next layers**

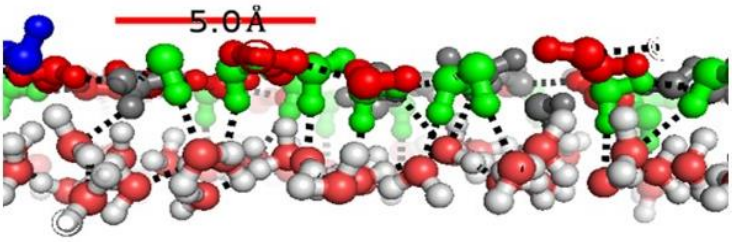

Fig. S5 Side view on interlayer HBs between interfacial (top) and next (lower) layers of water confined within graphite nanoslit of width 20Å. The molecular configuration was generated from our MD simulation. In the interfacial layer, molecules are specified as parallel-like (red), vertical-like (green), dangling-OH (blue), and others (gray). In the next layer, the red and gray spheres are O- and H-atoms of water molecule, respectively. The black dot lines indicate HBs between molecules.

● HB-configurations of layers within graphite nanoslits

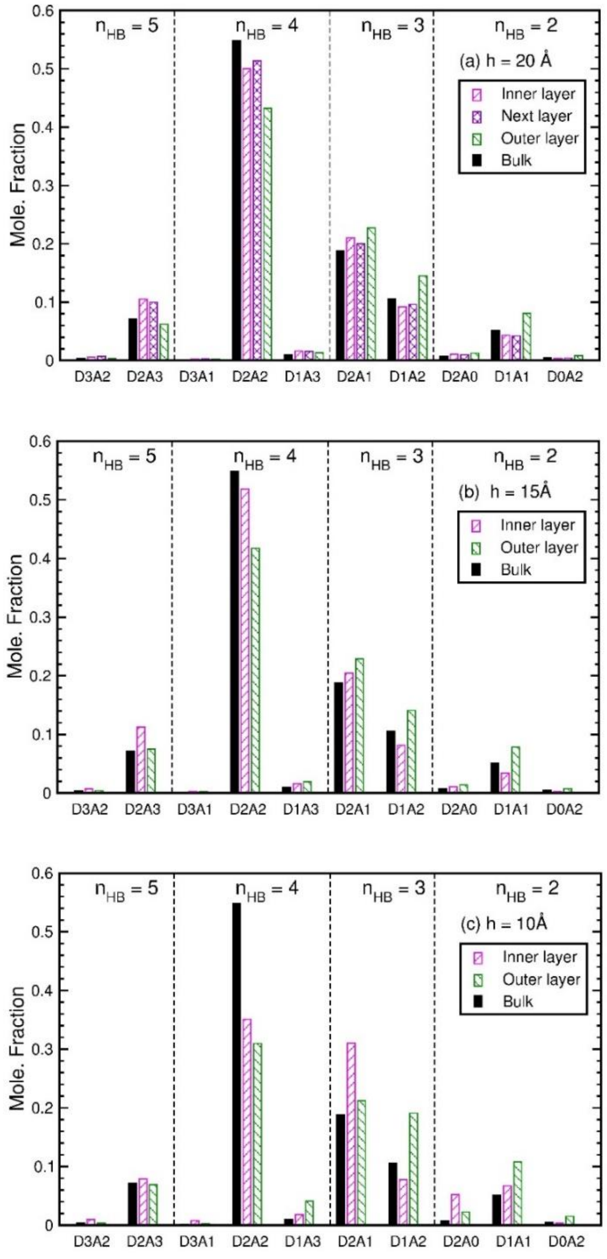

Fig. S6 Fractions of HB-configuration calculated for each layer within a graphite nanoslit of width: (a) $h = 20$Å, (b) $h = 15$Å, (c) $h = 10$Å. The fractions are ratios of DnAm molecules within a layer, where DnAm denotes a molecule having n donating and m accepting HBs within a neighborhood of the O-O distance less than 3.5 Å,[10] with the geometric HB-definition used.[11] For each layer, the fractions of HB-configuration are shown by bars of the same color and are normalized to one. In (b) and (c), nanoconfined water has outer and inner layers; in (a), a next layer is intermediate between outer and inner layers. The black bars are for TIP4P/2005 liquid water at ambient conditions.

● Orientational trajectories of interfacial molecules

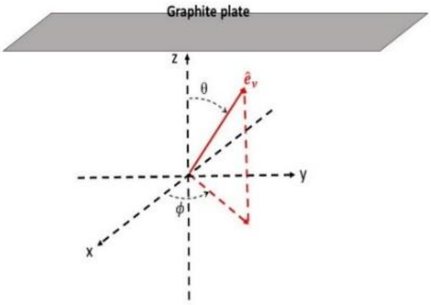

Fig. S7 Spherical polar coordinates (θ, ϕ) of unit vector $\hat{e}_v(t)$ relative to a C-atom plate. The polar angle θ is measured from the positive z-axis pointing to the plate and the azimuth angle ϕ is measured counterclockwise from x-axis in a plane parallel to the plate as viewing downward from the plate.

A. Trajectories at short times

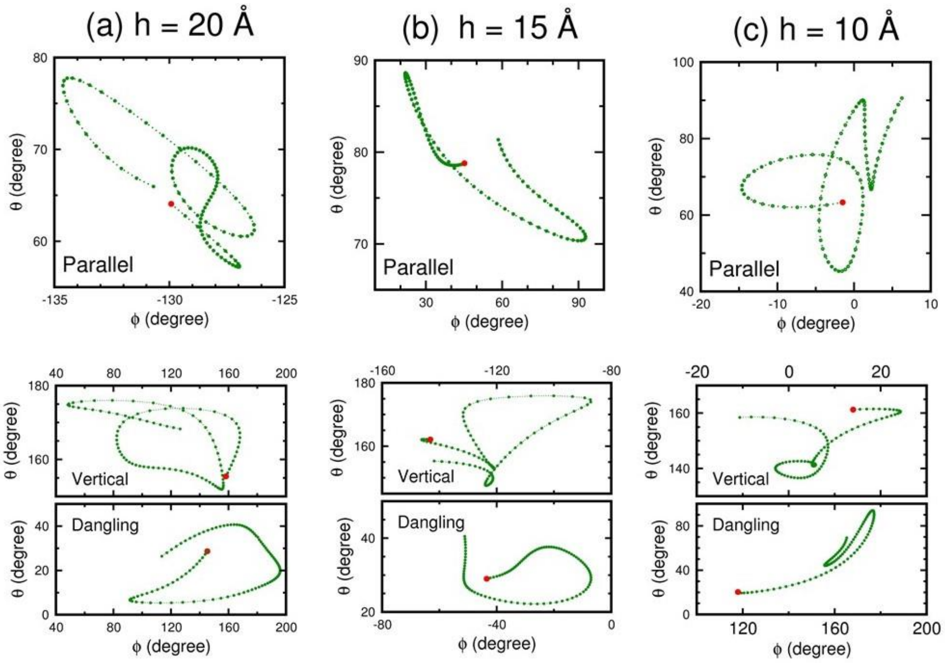

Fig. S8 Orientational trajectories of interfacial molecules up to 0.1 ps. The upper panels are for $\hat{e}_B$ of parallel-like molecules and the lower panels are for $\hat{e}_{OH}$ of vertical-like and dangling-OH molecules. The trajectories are plotted with spherical polar coordinates (θ, ϕ) of a unit vector shown in Fig. S7, and the data were obtained from our MD simulations. The red dots indicate the initial orientation and the filled green circles are points of trajectory at every 1 fs, guiding the eye with a small-dot line. In each plot, the local maxima and local minima of a trajectory are the turning points in θ, and the points with an infinite slope indicate a reverse in the direction of ϕ.

B. Trajectories at long times

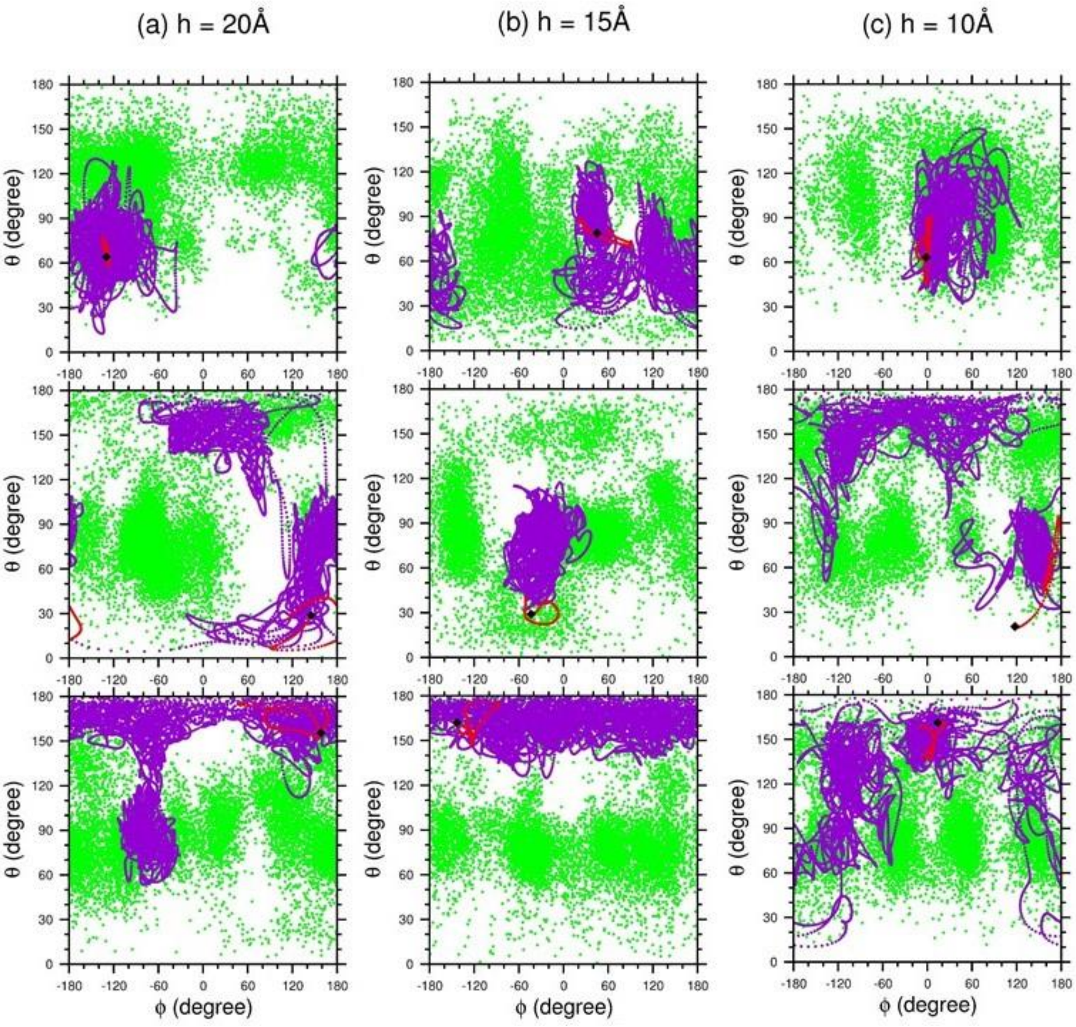

Fig. S9 Orientation trajectories of interfacial molecules at long times. The columns from left to right are for nanoslits of width 20Å, 15Å, 10Å, respectively. The top panels are for $\hat{e}_B(t)$ of parallel-like molecule. The middle and bottom panels are for $\hat{e}_{OH}(t)$ of dangling and vertical-like molecules, respectively. In each panel, the trajectory is plotted with spherical polar coordinates (θ, ϕ) of a unit vector, where trajectories with small θ indicate the unit vector points to a nearby confining wall. The black circle indicates the initial orientation of a unit vector. The red and violet circles present the trajectories from initial to 0.1 ps and from 0.1 to 10 ps, respectively, with points at every 1 fs shown. The green circles present the trajectory from 10 to 100 ps, with points at every 10 fs shown, so that the violet and green circles in each plot are equal in number.

● OTCFs and OVHFs of water layers within graphite nanoslits

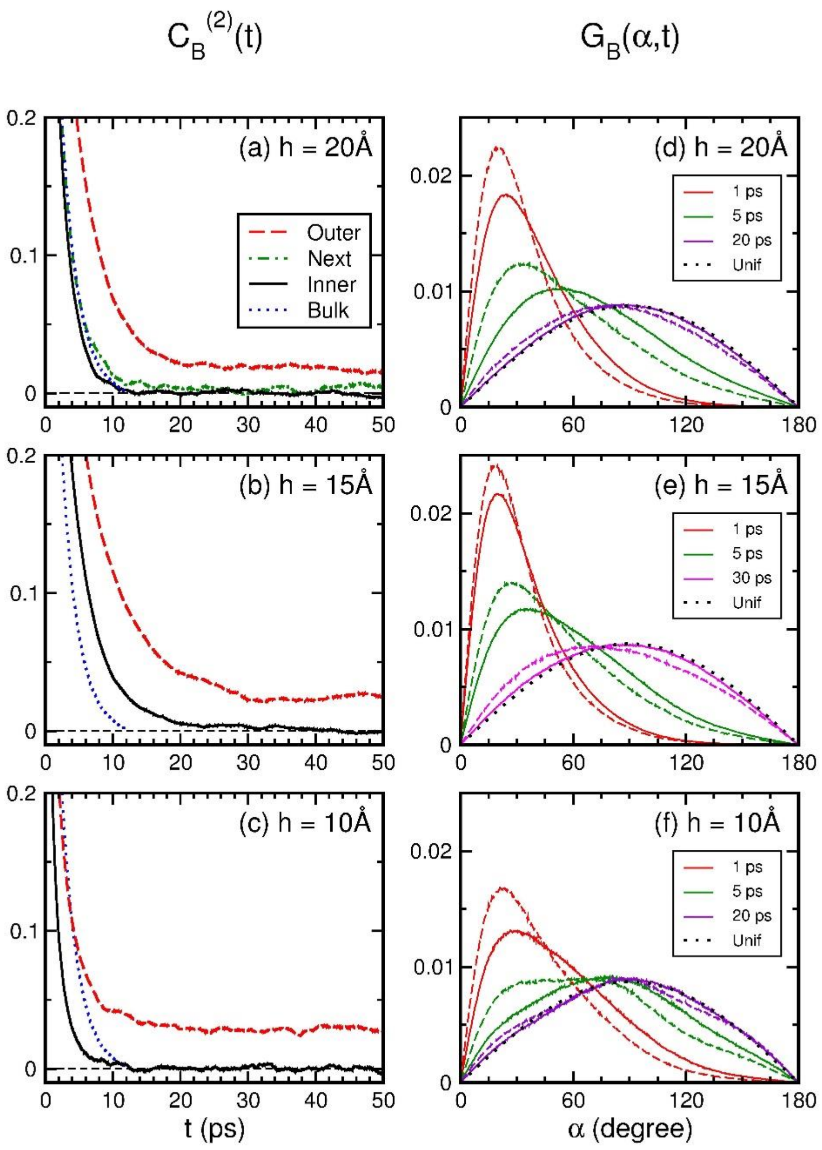

Fig. S10 OTCF $C_B^{(2)}(t)$ (left column) and OVHF $G_B(\alpha, t)$ (right column) of water layers within a graphite nanoslit. $C_B^{(2)}(t)$ and $G_B(\alpha, t)$ were calculated with the unit vector $\hat{e}_B(t)$ along a molecular dipole. The panels from top to bottom are for nanoslits of width 20Å, 15Å, 10Å, respectively. The left column is the second-rank OTCF $C_B^{(2)}(t)$ up to 50 ps, with black-solid, green-dot-dash, and red-dash lines for inner, next, and outer layers within a nanoslit, respectively. The blue-dot lines are for TIP4P/2005 liquid water at ambient conditions. The right column is OVHF $G_B(\alpha, t)$, with solid and dash lines for inner and outer layers within a nanoslit, respectively. The red, green, and violet (magenta) lines are OVHFs at 1, 5, and 20 (30) ps, respectively. The black dot lines are the OVHF of free molecules in random orientations.

■ References:

(1) Kuo, Y. W.; Wang, C. W.; Tang, P. H.; Wu, T. M. Layer structure and intermolecular vibrations of water confined within graphite nanoslits. *Chem. Phys. Lett.* **2023**, 825, 140612.

(2) Plimpton, S. Fast Parallel Algorithms for Short-Range Molecular Dynamics. *J. Comput. Phys.* **1995**, 117, 1.

(3) Abascal, J. L. F.; Vega, C. A general purpose model for the condensed phases of water: TIP4P/2005. *J. Chem. Phys.* **2005**, 123, 234505.

(4) Steele, W. A. *The Interaction of Gases with Solid Surfaces*; Pergamon: Oxford, 1974.

(5) Hansen J. P.; McDonald, I. R. *Theory of Simple Liquids*; Academic Press: New York, 2006.

(6) Allen, M. P.; Tildesley, D. J. *Computer Simulation of Liquids*; Oxford University Press: New York, 1987.

(7) Nosé, S. A. A unified formulation of the constant temperature molecular dynamics method. *J. Chem. Phys* **1984**, 81, 511.

(8) Hoover, W. G. Canonical dynamics: equilibrium phase-space distributions. *Phys. Rev. A* **1985**, 31, 1695.

(9) Giovambattista, N.; Debenedetti, P. G.; Rossky, P. J. Effect of surface polarity on water contact angle and interfacial hydration structure. *J. Phys. Chem. B* **2007**, 111, 9581-9587.

(10) Chang, S. L.; Wu, T. M.; Mou, C. Y. Instantaneous normal mode analysis of orientational motions in liquid water: Local structural effects. *J. Chem. Phys.* **2004**, 121, 3605-3612.

(11) Luzar, A.; Chandler, D. Hydrogen-bond kinetics in liquid water. *Nature* **1996**, 379, 55-57.

(12) Tang, P. H.; Fan, Y. Y.; Hsu, W. L.; Wu, T. M. Reorientation of OH-group connecting bifurcated H-bond acceptors in liquid water. *Chem. Phys. Lett.* **2018**, 710, 168-174.
